# Microstructure and Cerebral Blood Flow within White Matter of the Human Brain: A TBSS Analysis

**DOI:** 10.1371/journal.pone.0150657

**Published:** 2016-03-04

**Authors:** Stéphanie Giezendanner, Melanie Sarah Fisler, Leila Maria Soravia, Jennifer Andreotti, Sebastian Walther, Roland Wiest, Thomas Dierks, Andrea Federspiel

**Affiliations:** 1 Center for Translational Research, University Hospital of Psychiatry and Psychotherapy, University of Bern, Bern, Switzerland; 2 University Hospital of Psychiatry and Psychotherapy, University of Bern, Bern, Switzerland; 3 Support Center for Advanced Neuroimaging (SCAN), Institute for Diagnostic and Interventional Neuroradiology, University Hospital Inselspital and University of Bern, Bern, Switzerland; University of Pennsylvania, UNITED STATES

## Abstract

**Background:**

White matter (WM) fibers connect different brain regions and are critical for proper brain function. However, little is known about the cerebral blood flow in WM and its relation to WM microstructure. Recent improvements in measuring cerebral blood flow (CBF) by means of arterial spin labeling (ASL) suggest that the signal in white matter may be detected. Its implications for physiology needs to be extensively explored. For this purpose, CBF and its relation to anisotropic diffusion was analyzed across subjects on a voxel-wise basis with tract-based spatial statistics (TBSS) and also across white matter tracts within subjects.

**Methods:**

Diffusion tensor imaging and ASL were acquired in 43 healthy subjects (mean age = 26.3 years).

**Results:**

CBF in WM was observed to correlate positively with fractional anisotropy across subjects in parts of the splenium of corpus callosum, the right posterior thalamic radiation (including the optic radiation), the forceps major, the right inferior fronto-occipital fasciculus, the right inferior longitudinal fasciculus and the right superior longitudinal fasciculus. Furthermore, radial diffusivity correlated negatively with CBF across subjects in similar regions. Moreover, CBF and FA correlated positively across white matter tracts within subjects.

**Conclusion:**

The currently observed findings on a macroscopic level might reflect the metabolic demand of white matter on a microscopic level involving myelination processes or axonal function. However, the exact underlying physiological mechanism of this relationship needs further evaluation.

## Introduction

Brain white matter (WM) consists of axons connecting neurons and of neuroglial cells that support and protect neurons [1]. The myelination of axons provides an electrical insulation that enhances the impulse conduction and supports axonal integrity [2–4]. Intact white matter microstructure appears crucial for proper brain functioning and is altered in psychiatric disorders and neurodegenerative diseases [5–9]. However, little is known about the perfusion of white matter, e.g. the cerebral blood flow, and how it is related to the microstructure of white matter [10, 11].

Magnetic resonance imaging (MRI) enables the assessment of microstructure and physiology of white matter non-invasively and in vivo [12]. On the one hand, diffusion tensor imaging (DTI) offers a measure of different white matter microstructural properties [13]. On the other hand, arterial spin labeling (ASL) is used to quantify cerebral blood flow (CBF) in the human brain [14]. Yet, the measurement of blood perfusion in WM with ASL was claimed to be challenging since the blood perfusion of white matter is lower, more heterogeneous and the ASL labelled bolus takes longer to arrive and therefore the T1 decay of the signal is more advanced than that of grey matter (GM) [10, 15, 16]. However, with the implementation of pseudo-continuous ASL (pCASL) at 3T, the signal-to-noise (SNR) ratio as well as the efficiency of the tagging was shown to be increased [17]. Recent studies indicate that white matter CBF can be reliably detected using pCASL at 3T [15, 16].

Regarding the relationship between cerebral blood flow and white matter microstructure, clinical studies provide accumulating evidence that WM health is closely related to its perfusion with blood. In Alzheimer’s disease and multiple sclerosis, reduced brain perfusion was associated with an increase in WM lesions, hyperintensities or decreased anisotropic diffusion across subjects [18, 19]. Additionally, cerebrovascular diseases were related to decreased anisotropic diffusion, white matter hyperintensities and cognitive decline across the elderly [20–25]. These findings suggest that certain WM regions might be particularly vulnerable to hypoperfusion due to its blood supply via long penetrating arterioles [26]. In summary, pathologies which impede proper brain perfusion tend to affect WM health.

However, little is known about white matter blood perfusion and its relation to white matter microstructural properties across healthy subjects and across fiber tracts. Notably, neuroanatomic studies of the brain vascular system indicate that blood supply patterns in white matter are regionally different, explaining the variations in vulnerability to perfusion or oxygen deficiency [27]. Moreover, white matter fibers vary in size and density according to their function, activation and location within the brain [28–32]. Recent studies indicate that WM maintenance is an active and energy-demanding process [33, 34]. Considering the above mentioned relationship between WM health and cerebral perfusion and the physiological properties of WM, we hypothesized to find a positive correlation between measures of WM anisotropic diffusion properties and WM perfusion. Intriguingly, Aslan and colleagues reported an inverse relationship between anisotropic diffusion and WM perfusion on a tract-specific basis within subjects [11]. In particular, tracts with higher anisotropic diffusion were shown to be less perfused [11]. However, this study investigated a limited number of WM tracts using tractography and averaged diffusion metrics along the fiber tracts. Thus, the specific regional relationship between WM integrity and WM perfusion across subjects has not yet been assessed conclusively.

As a consequence, the current study aimed to probe for the relationship between WM perfusion and WM microstructural properties across subjects. We hypothesized that CBF in white matter is positively related to anisotropic diffusion of water across healthy subjects. To test this hypothesis, we implemented a voxel-wise analysis approach to investigate the whole WM skeleton across subjects [35]. Furthermore, we aimed to assess the tract-specific relationship between CBF and anisotropic diffusion of water within subjects [11]. According to the study of Aslan and colleagues (2011), we hypothesized to find an inverse correlation between WM perfusion and anisotropic diffusion across fiber tracts within subjects. To test this hypothesis, we performed an atlas-based ROI analysis within subjects.

## Materials and Methods

### Participants

A total of 43 right-handed subjects were studied (15 men and 28 women) with an age range between 20 and 46 years (mean = 26.30, SD = 5.29 years). Procedures were approved by the local ethics committee (of the Kanton Bern, Switzerland) and are in accordance with the Declaration of Helsinki. The full KEK Commission Board Bern specifically approved this study and assigned the KEK-Nr: 196–09. All participants provided written informed consent prior to the examination. Subjects were not paid for participation. The Exclusion criteria included contraindications for MRI scanning (metallic objects, pregnancy), acute or chronic medical condition, neurological or severe psychiatric diseases, current drug or alcohol abuse, regular medication or contraceptives.

### MRI Data Acquisition

Magnetic resonance imaging was performed on a 3 T Siemens Magnetom Trio Scanner (Erlangen, Germany) equipped with a standard radio-frequency head coil.

#### Anatomical high-resolution T1-weighted sequence

For structural images, a high-resolution 3D T1-weighted imaging protocol modified driven equilibrium Fourier transform (MDEFT) [36] was used, resulting in 176 sagittal slices of 1.0 mm thickness, 256 × 256 mm^2^ field of view (FOV), and a matrix size of 256 × 256, resulting in a voxel size of 1 × 1 × 1 mm^3^. Further scan parameters were 7.92 ms repetition time (TR) 2.48 ms echo time (TE) and 910 ms inversion time (TI) for an optimized contrast-to-noise ratio [36].

#### DTI data acquisition

Diffusion tensor imaging encompassed the following parameters: FOV = 256 × 256 mm^2^, matrix = 128 × 128, axial slices = 55, slice thickness = 2 mm, gap = 0 mm, voxel size = 2 × 2 × 2 mm^3^, TE/TR = 93/7500 ms. DTI was performed with a spin echo planar imaging (EPI) using two 180° pulses at b-value of 0 s/mm^2^ and at a maximal b-value of 1300 s/mm^2^ along 42 non-collinear directions.

#### pCASL MRI data acquisition

For the measurement of CBF a pseudo continuous arterial spin labeling (pCASL) technique with the following parameters was used [17, 37]: TR/TE = 3500/18 ms, FOV = 230 × 230 mm^2^, matrix size = 64 × 64 (Isocenter of the readout slice was 90 mm above labeling plane), balanced labeling with mean Gz of 0.6 mT/m and 60 Hanning window-shaped RF pulses (RF duration 600 μs with 900 μs gap, a flip angle (FA) = 25°, and bandwith 3,004 Hz/pixel), total labeling duration (*τ*) = 1.72 s and post-labeling delay (ω)(PLD) = 1.1 s. 13 axial slices, voxel size = 3.6 x 3.6 x 6.5 mm^3^, 6.5 mm slice thickness and 0.5 mm gap. All ASL and DTI slices were positioned along the AC-PC line and were recorded (Siemens Erlangen, Germany). The number of timeseries (pairs of control and labeling images) varied between subjects (see S5 and S6 Figs). In order to assess the quality of the signal in WM across PLD we additionally recorded five subjects with the same ASL setting with three different PLD values: 1100 ms, 1525 ms (according to [11]) and 1900 ms (according to “ASL white paper” [38]). SNR and CBF values were subsequently compared across PLDs (see S16 and S17 Figs).

### MRI Data Analysis

#### DTI data processing

Preprocessing of the FA data was carried out using TBSS [35], part of FSL 5.0 [39]. First, diffusion images were corrected for head movement and eddy currents. Then, FA images were created by fitting a tensor model to the raw diffusion data using FDT and then brain-extracted using BET [40]. All subjects' FA data were then aligned into 1 × 1 × 1 mm MNI space using the nonlinear registration tool FNIRT [41, 42], which uses a b-spline representation of the registration warp field [43]. Next, the mean FA image was created and thinned to create a mean FA skeleton representing the centers of all tracts common to the group. This skeleton was then thresholded at a FA value of 0.3 in order to minimize partial voluming effects. Each subject's aligned FA data was then projected onto this skeleton and the resulting data fed into voxel-wise cross-subject statistics. Additionally to the FA images, further putative indices of tissue microstructure were calculated such as the mean diffusivity (MD), radial diffusivity (RD) and axial diffusivity (AD).

#### Partial volume estimates based on anatomical high-resolution T1-weighted sequence

The intensities of the three-dimensional T1-weighted scans were used to automatically segment brain tissue into cerebrospinal fluid (CSF), GM and WM by a robust method treating the partial volume affected voxels as outliers of the pure tissue distribution [44]. This trimmed minimum covariance determinant method enabled a rigorous treatment of the partial volume estimates (PVE) in each voxel [45]. For the creation of GM and WM masks, the respective tissue was subtracted from all other contaminations, resulting in GM or WM masks consisting of 100% GM or WM tissue respectively. The resulting GM and WM masks were then transformed into ASL space. Finally these masks were thresholded at 40% and 70% respectively to generate binary masks for the use in subsequent analyses (see S1 Fig and Partial volume correction).

#### ASL data processing

First, the raw pCASL data were motion-corrected using MCFLIRT in FSL 5.0 [46], which loads the time-series and uses the middle volume as an initial template image (see S3 Fig for motion parameter estimation). Control-tag pairwise differences were computed to yield a mean difference map for each subject. These difference maps were normalized by the equilibrium magnetization (*M*_*0*_) of arterial blood estimated via the magnetization of CSF.

### CBF quantification

For the quantification of CBF a variational Bayesian approach accounting for the differences in acquisition time across the slices as implemented in FSL 5.0 [47] was used (BASIL). The calibration of ASL images in order to obtain CBF maps in absolute units of ml/100g/min was performed using the following equation.

$$CBF=\left(\frac{\lambda \cdot \Delta M}{2\cdot \alpha \cdot M_{0}\cdot T_{1b}}\right)\cdot \left(\frac{1}{e^{-w/T_{1b}}-e^{-\left(\tau +w\right)/T_{1b})}}\right)$$

Post-labeling delay time (*ω*) = 1100 ms, bolus duration (*τ*) = 1720 ms, the blood tissue partition coefficient (*λ*) = 0.9 [g/ml] [48] and the inversion efficiency assumed to be (*α*) = 85%. Δ*M* is the difference between the control and the label signal. For 3.0 Tesla the decay time for the labeled blood (*T1b*) = 1650 ms, the tissue longitudinal relaxation time of white matter = 1084 ms [49]. *M*_*0*_ are the equilibrium magnetization images, which were first calculated for CSF and were then converted to the equilibrium magnetization of arterial blood. Furthermore, in the kinetic model the bolus arrival time (BAT) was set to 1300 ms. Moreover, variance maps of CBF were created to allow for the assessment of uncertainty of estimation in WM, which was passed up into higher-level statistical analysis.

The signal-to-noise (SNR) calculation with the PVE corrected CBF images were done as described in Aslan et al [50] using as the signal level the CBF image and as the noise level the CBF variance image. The latter was used to calculate the standard error and was divided by the square root of the number of pairs (in our experiment this was 50, 100, 150 or 200). More in detail, the SNR was calculated for each voxel and for each subject. It served as a basis to calculate the SNR in different ROI’s (e.g. WM and GM masks). SNR was averaged over all the voxels for each of these different masks separately.

#### Partial volume correction

The low resolution of ASL data typically means that there is partial voluming of GM, WM and CSF. Since GM and WM have varying kinetics–with WM having a lower CBF and longer arrival times–a normal analysis will provide a CBF that are ambiguous (e.g. a combination of the two tissue types). Thus, the contributions of different tissue types were corrected for by exploiting PVE as obtained from high resolution T1 images (see Partial Volume Estimates based on Anatomical High-resolution T1-weighted Sequence above) that were transformed to same resolution of ASL images [51, 52]. The resulting CBF images are therefore corrected for possible contributions from partial volume effects.

#### Multimodal data and fusion

Inter-scan registrations were conducted using FLIRT (FMRIB's Linear Image Registration Tool, www.fmrib.ox.ac.uk/fsl) of FSL 5.0. FLIRT is a fully automated robust and accurate tool for linear (affine) intra- and inter-modal brain image registration [46, 53]. For the statistical analysis, the CBF maps, the perfusion variance and the SNR maps were registered to the FA images using an affine 12 parameter model provided by the FLIRT algorithm. As mentioned in 2.3.1. GM probability maps were co-registered to the FA images using an affine 12 parameter model provided by the FLIRT algorithm. The skeletons of the CBF, SNR, perfusion variance and GM maps were obtained by applying the non-FA option in TBSS including the nonlinear warps and skeleton projection from the FA maps to these maps.

#### Statistical analysis

Statistical analysis was carried out with FSL Randomise program, which is based on a non-parametric approach using permutation test theory with a standard general linear model (GLM) design matrix [54, 55]. The GLM examined the association of microstructural properties such as FA, MD, AD and RD values with CBF values on a voxel-wise basis in the WM skeleton across subjects. Covariates such as age, gender the number of ASL time series and mean relative displacement were entered into the analyses as explanatory variables, while individual maps of variance in the ASL data, maps of the partial voluming of grey matter and CBF maps were entered as voxel-dependent regressors into the model [56]. All covariates were demeaned before running the randomise program. The permutation testing was performed using 5,000 Monte Carlo random permutations to generate random permutation maps. This approach allows for inference on the statistical maps when the null distribution is unknown and offers a solution to the problem of multiple testing. A threshold-free cluster enhancement (TFCE) option was employed in order to enhance the cluster-like structures in images without having to define an initial cluster-forming threshold [57]. Using this setup the voxel-wise relation between microstructural properties and CBF values across subjects were then assessed, testing the significance corrected for multiple comparisons (TFCE corrected *p* < 0.05) or uncorrected for multiple comparisons (TFCE uncorrected *p* < 0.05). From the results of the voxel-wise analyses, the skeletal regions showing significant effects were located and labelled anatomically by mapping the statistical map to the John Hopkins University (JHU)-ICBM-DTI-81 WM labels atlas and the JHU-WM tractography atlas in MNI space [58, 59]. In the tables, only clusters greater than 50 voxels per WM region were shown. For the comparison of the relationship between CBF and FA across different fiber tracts within subjects, the mean of the FA and CBF values across the different WM labels and tracts of the above mentioned John Hopkins University (JHU) atlases were extracted.

For visualization purposes we use the FSL “tbss_fill” command: a command that visualizes the results not on the TBSS skeleton (i.e. the true representation of actual analysis) but on a “thickened” skeleton (i.e. convolution of the TBSS skeleton with a sphere of radius 3 mm).

#### Descriptive statistics

For comparison purposes, the mean values of diffusion and perfusion measures were extracted in GM and WM across subjects and listed in Table 1. The creation of GM and WM masks is described in the supplementary material (see S1 Fig). Four subjects who presented particularly low CBF in GM (less than 38 ml/100g/min) were excluded from the subsequent statistical analyses (see S4 Fig). The six motion parameters at each time point were converted to a time course measure of the relative RMS voxel displacement. Finally, the temporal average of this time course displacement signal was used to represent overall subject motion for the session. This metric is termed the mean relative displacement (MRD), and is expressed in mm (see S3 Fig). This metric was used as a covariate in the statistical analyses.

**Table 1:** Summary of mean ± standard deviations in WM and GM of FA, AD (μm^2^/ms), MD (μm^2^/ms), RD (μm^2^/ms), CBF, SNR and ASL signal (%) across subjects (*n* = 43).

	WM	GM
**FA**	0.47±0.02	0.29±0.01
**AD** (μm^2^/ms)	1.03±0.03	1.03±0.06
**MD** (μm^2^/ms)	0.68±0.03	0.83±0.05
**RD** (μm^2^/ms)	0.50±0.03	0.72±0.05
**CBF** (ml/100g/min)	19.7±2.7	48.1±8.9
**SNR**	2.9±0.77	4.22±1.26
**Significant ASL signal** (%)	66.5±12.2	75.8±8.4

## Results

### Descriptive results of microstructural and ASL-related properties

Table 1 summarizes CBF, FA, AD, RD, MD and SNR of CBF as well as the proportion of significant ASL signal in the WM and GM across subjects (see Methods of Supplementary Material). Notably in WM, the mean CBF was found to be 19.7 ml/100g/min with a mean signal to noise ratio of 1.33 and a mean proportion of voxels with a significant ASL signal of 66.5%. The latter indicates that in 66.5% of WM voxels the difference of the control and label image was significantly different from zero and thus the ASL signal was reliably detected [15]. In the supplementary material, the proportion of significant ASL signal in WM and GM is displayed within one subject across 200 ASL signal averages and across subjects with differing numbers of averaged ASL signal (see S5 and S6 Figs). Moreover, S2 Fig shows the SNR and the CBF in GM and WM as well as the GM and WM masks along the axial slice of a subject. S3 Fig shows the mean relative displacement as the relative voxel displacement RMS in mm across the ASL timeseries. S7 Fig displays the effects of the different numbers of averages on CBF and SNR in GM and WW.

### Correlation of microstructural properties with CBF across subjects

**Fig 1:**
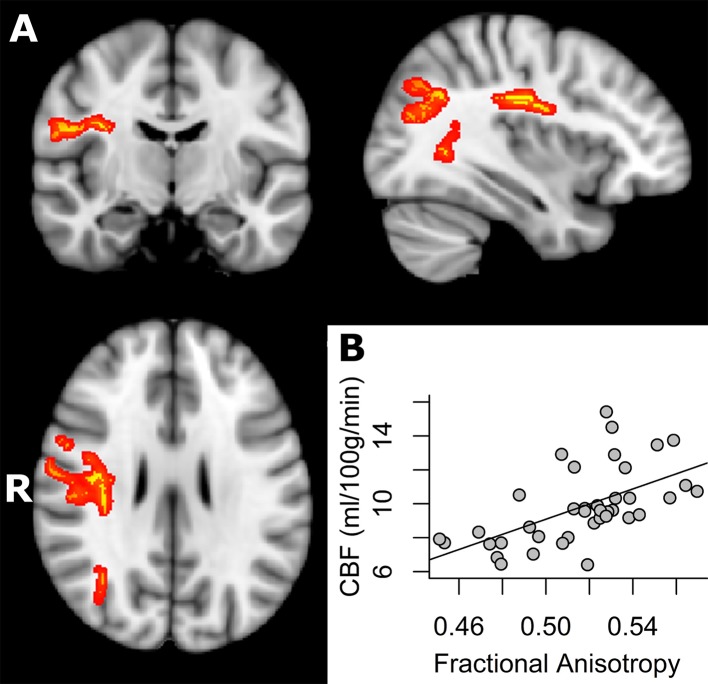
A) Regions of significant positive correlation between CBF and FA across subjects overlaid on the MNI template (in grey) at x = 54, y = 115, z = 99 (TFCE corrected *p* < 0.05). Regions of significant positive correlation between CBF and FA across subjects are indicated in red (tbss_fill was used here which “thickened” the TBSS results). B) The scatterplot shows the mean FA and CBF values extracted from each subject in the significant regions indicated in.

The statistical analyses probing for a relationship between CBF and white matter properties across subjects were co-varied for age, gender, the number of ASL time series, mean relative displacement (MRD), the variance in the ASL data and the partial volume effects of grey matter. Results of the TBSS analysis showed a significant positive correlation (TFCE corrected *p* < 0.05) between fractional anisotropy and CBF values across subjects in parts of the splenium of corpus callosum, the right posterior thalamic radiation (include optic radiation), the forceps major, the right inferior fronto-occipital fasciculus, the right inferior longitudinal fasciculus and the right superior longitudinal fasciculus (Fig 1, Table 2). Notably, the SNR in the brain regions displayed in Fig 1 is significantly greater than 2.0 for CBF values [t(38) = 6.25; p < 0.0001; see S14 Fig].]. Furthermore, the difference of control and label image within the brain regions displayed in Fig 1 is significantly greater than 2.0 [t(38) = 2.75; p < 0.005; see S15 Fig]. The positive relationship between CBF and FA across subjects is additionally displayed for each WM region separately in S8 Fig. The proportion of voxels displaying a significant ASL signal within the significant cluster of this analysis was 70.1%.

**Table 2:** Summary of the regions including volume (mm), FA, CBF (ml/100g/min) and *t* values where a significant positive relationship between CBF and FA values was found across subjects. The values were extracted by masking the respective skeletons with the JHU WM labels and tracts and the significant regions (TFCE corrected *p* < 0.05). The values are listed as mean ± standard deviation.

	Volume	CBF	FA	*T* values
Forceps major	243	9.22±6.77	0.70±0.14	2.52
Inferior fronto-occipital fasciculus R	248	8.83±7.15	0.60±0.11	2.37
Inferior longitudinal fasciculus R	79	8.07±6.76	0.49±0.12	2.29
Superior longitudinal fasciculus R	1351	10.69±8.62	0.48±0.11	2.18
Splenium of corpus callosum	122	9.29±6.54	0.78±0.12	2.47
Posterior thalamic radiation (include optic radiation) R	239	8.94±6.99	0.64±10	2.47

**Fig 2:**
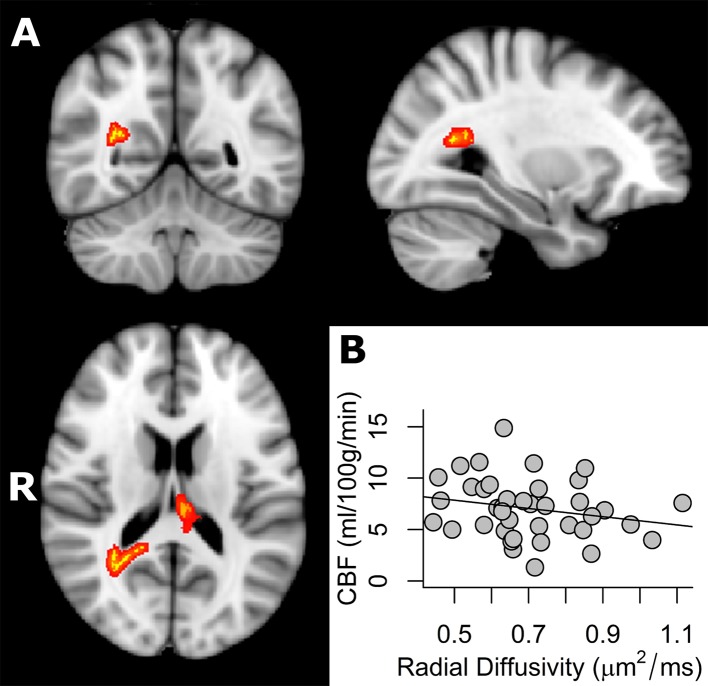
A) Regions of significant negative correlation between CBF and RD values overlaid on the MNI template at x = 60, y = 72, z = 89 (TFCE corrected *p* < 0.05). Regions of significant positive correlation between CBF and RD across subjects are indicated in red (tbss_fill was used here which “thickened” the TBSS results). B) The plot displays the mean RD and CBF values, extracted from each subject in the significant regions indicated in red.

In addition, TBSS analysis demonstrated a significant negative correlation between CBF and radial diffusivity (TFCE corrected *p* < 0.05) in parts of the splenium of corpus callosum, the right posterior thalamic radiation (include optic radiation) and the forceps major (Fig 2 and Table 3). When not correcting for multiple comparisons, CBF was significantly correlated with FA (positively), RD (negatively) and MD (negatively) within broad areas of the whole WM skeleton (TFCE uncorrected *p* < 0.05) (see S9, S10 and S11 Figs).

**Table 3:** Summary of the regions including volume (mm), RD (μm^2^/ms), CBF (ml/100g/min) and *t* values where a significant negative relationship between CBF and RD values was found across subjects. The values were extracted by masking the respective skeletons with the JHU WM labels and tracts and the significant regions (TFCE corrected *p* < 0.01). The values are listed as mean ± standard deviation.

Locations	Volume	CBF	RD	*T* values
Splenium of corpus callosum	85	7.65±6.80	0.48±0.38	2.63
Posterior thalamic radiation (include optic radiation) R	69	9.08±7.10	0.42±0.08	3.09
Forceps major	116	8.71±6.98	0.43±0.34	2.72

**Fig 3:**
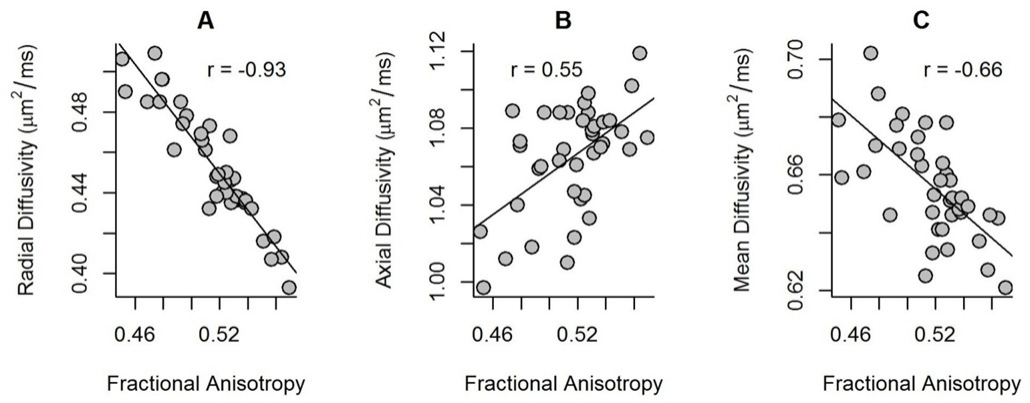
The scatterplot displays the mean RD, AD and MD as a function of the mean FA values across subjects, all extracted from the significant regions indicated in red in Fig 1. A) The correlation between FA and RD was negative (Pearson’s *r* = -0.93, *p* < 0.0001). B) The correlation between FA and AD was positive (Pearson’s *r* = 0.55, *p* < 0.001). C) The correlation between FA and MD was negative (Pearson’s *r* = -0.66, *p* < 0.0001).

In addition, Fig 3 indicates the relationship between mean FA and mean RD, MD and AD within the regions that showed a significant positive correlation between microstructural properties and cerebral blood flow values across subjects (Fig 1). The mean values in Fig 3 were all extracted from the regions of a significant positive correlation between mean FA and mean CBF values across subjects.

### Correlation of microstructural properties with CBF across tracts

**Fig 4:**
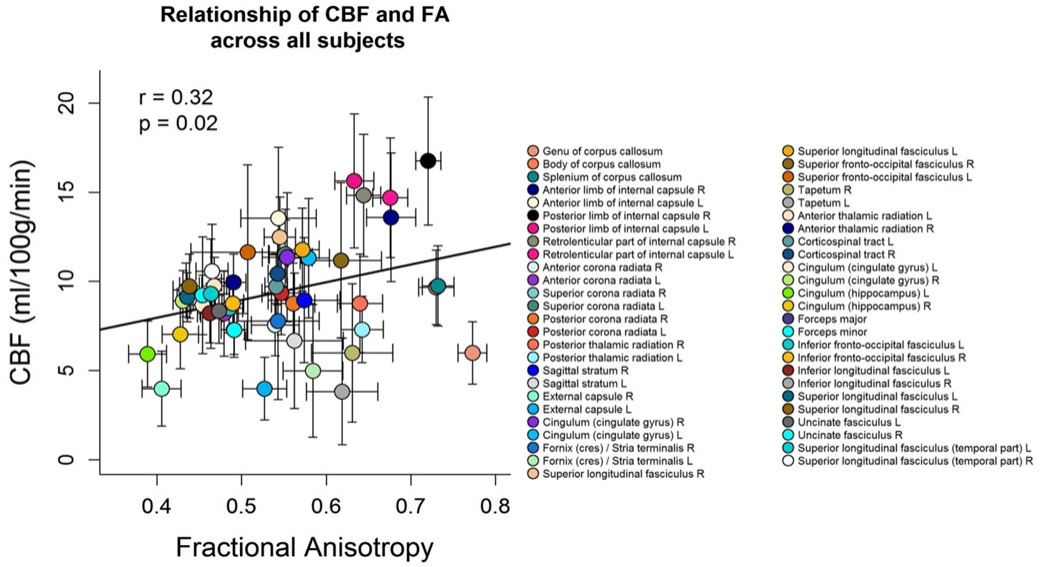
Scatterplot between FA and CBF values. Each symbol represents data from one WM region averaged over all subjects. The error bars indicate the standard deviation over all subjects. The mean FA over all subjects was positively correlated with mean CBF of all subjects across WM regions (Pearson’s *r* = 0.32, *p* = 0.02). The legend on the right side refers to the different WM regions.

Fig 4 shows the scatter plot between FA and CBF for the group-averaged data. Each symbol represents data from one WM region averaged over all subjects. The mean FA values of the 51 different WM regions were positively correlated with the mean CBF values across subjects (Pearson’s *r* = 0.32, *p* = 0.02). High FA (> 0.6) and high CBF (> 12 ml/100g/min) were found in tracts containing projection fibers which are characterised by mainly large diameter fibers and fast conduction velocity such as the right anterior limb of internal capsule, the bilateral posterior limb of internal capsule and the bilateral retrolenticular part of internal capsule. The internal capsule contains both ascending and descending projection fibers that connect the cortex with the spinal cord, such as the corticospinal tract which carries motor information from the primary motor cortex to the lower motor neurons in the spinal cord [60]. Low FA (< 0.5) and low CBF (< 8 ml/100g/min) were found in long association fibers characterized by mainly small diameter fibers such as the right external capsule, the bilateral cingulum (hippocampus), the forceps minor and major [61]. The external capsule connects cortical areas with the putamen, caudate nucleus, and claustrum [28, 62]. The cingulum has fibers projecting from the cingulate gyrus to the entorhinal cortex in the brain, allowing for communication between components of the limbic system [28]. While the forceps minor connect the frontal lobe areas through the genu of the corpus callosum, the forceps major associates the occipital areas of both hemispheres through the splenium of the corpus callosum. Additional analyses within each subject showed that in 21 subjects out of 39 the association between CBF and FA across white matter regions was significantly positive (see S1 Table).

## Discussion

The current study presents the investigation of white matter cerebral blood flow and its relationship to white matter microstructural integrity across subjects, exploring CBF and DTI metrics (FA/MD/AD/RD). Since anisotropic diffusion measured with DTI was shown to be sensitive to barriers and obstacles imposed by microstructural properties such as cell membranes, myelin sheaths and microtubules, it was suggested to represent a putative surrogate of microstructural integrity (Beaulieu, 2002). Voxel-wise analysis demonstrated a positive association of white matter CBF and FA across subjects in circumscribed white matter regions. Thus, subjects who had higher CBF tended to have higher fractional anisotropy in parts of the splenium of corpus callosum, the right posterior thalamic radiation (include optic radiation), the forceps major, the right inferior fronto-occipital fasciculus, the right inferior longitudinal fasciculus and the right superior longitudinal fasciculus (Fig 1 and Table 2). Subsequent analyses demonstrated a negative correlation between CBF in white matter and RD in parts of the splenium of corpus callosum, the right posterior thalamic radiation (including the optic radiation) and the forceps major (but no relationship with MD or AD, when correcting for multiple comparisons) (Fig 2 and Table 3). These findings could not be explained by age, sex, number of ASL image averages, mean relative displacement (mm), voxel-wise variance in perfusion signal or voxel-wise partial voluming of grey matter. Furthermore, the tract-specific analysis of the relationship between FA and CBF within subjects showed that fiber tracts that had a higher CBF tended to have a higher anisotropic diffusion (Fig 4 and S1 Table).

Our voxel-wise analyses were intended to identify specific locations of a correlation between CBF and anisotropic diffusion of water in white matter tracts across subjects. In order to increase sensitivity, objectivity and interpretability of analysis of multi-subject diffusion imaging studies, correlation analysis was limited to an alignment-invariant tract representation (TBSS skeleton). Notably, the identified white matter regions indicated that especially posterior and right-hemispheric white matter structures were the regions with most voxels displaying a positive correlation (Fig 1, Table 2). However, the stringent corrections for multiple comparisons that are required in this type of analyses may have dismissed regions that showed a less strong association. Indeed, when not correcting for multiple comparisons, positive correlations between white matter CBF and FA as well as negative correlations between white matter CBF and RD across subjects were observed in widespread WM areas in both hemispheres (see S9 and S10 Figs). Furthermore, uncorrected results for multiple comparisons also showed that white matter CBF was negatively related to MD and AD in circumscribed regions (see S11 and S12 Figs). We thus suggest that the positive relationship between CBF and white matter microstructural integrity most probably does not only apply to the listed regions in Table 2, but might hold for the whole WM skeleton although to a lesser extent. Nevertheless, the association seems to be stronger in the right hemisphere, which might relate to known anatomic and functional lateralization of the human brain. There is evidence that functional differences in interhemispheric coordination might relate to the brain's hierarchical subdivisions [63]. Further, asymmetry of myelination was documented in posterior superior temporal lobes indicating a left hemisphere dominance for rapid sensory signal processing [64, 65]. Since we analysed right-handers with 90–95% probability of left hemispheric language dominance [66], the right hemisphere might display more inter-individual variance in microstructural and functional properties. Indeed, increased right hemispheric inter-individual variance was observed for FA in the current study and might have contributed to stronger associations between CBF and FA across subjects.

Since anisotropic diffusion is based on different microstructural white matter properties (Beaulieu, 2002), the current methods are not suited to disentangle the exact underlying microstructural properties that contributed to the observed positive relationship between anisotropic diffusion and CBF in white matter across subjects [67]. Nevertheless, different diffusion metrics such as RD, MD and AD may provide additional indications about the nature of the relationship between CBF and underlying microstructural characteristics in white matter. The positive association between white matter CBF and FA was primarily characterized by a negative correlation between white matter CBF and radial diffusivity, in the absence of a significant correlation with axial diffusivity or mean diffusivity. When plotting the mean FA values against the mean radial diffusivity, mean diffusivity and axial diffusivity which were extracted from the regions with a significant positive association between white matter CBF and FA across subjects, all values are significantly associated with FA (Fig 3). However, radial diffusivity was most strongly related to FA compared with mean diffusivity or axial diffusivity (Fig 3A). Intriguingly, the observation of increased radial diffusivity in absence of axial diffusivity alterations has been found in well controlled animal studies of experimentally induced myelin loss [68, 69]. Thus, the current results of a significant relationship between white matter perfusion and white matter microstructural integrity might be associated with myelination properties of white matter.

Little is known about the neurophysiological mechanisms that may underlie the observed relationship between CBF and microstructural integrity in white matter across subjects. Yet, recent findings on the metabolism of central nervous system white matter indicate that white matter is not only a passive insulator. On the first sight, myelin seems to be an energy-efficient mechanism, by speeding up the process of signal conduction with its insulating characteristics. However, the synthesis of myelin and the maintenance of oligodendrocyte resting potential were shown to outweigh the energy savings by saltatory impulse propagation [70]. Recent studies indicate that the axonal energy metabolism is supported by glycolytic oligodendrocytes [34, 71]. The exact mechanism is not yet fully understood but it seems that oligodendrocytes might provide the axons with metabolites across the soma and cytoplasm near the periaxonal space underneath the myelin sheath. Furthermore, the role of lactate release from oligodendrocytes was found to be essential for long-term axonal integrity [34]. In summary, recent evidence indicates that myelinating glial cells enable preservation of long-term axonal integrity by detection of axonal energy needs and providing the necessary metabolites.

Interestingly, myelination is affected by the underlying neuronal electrical activity and increases with the activity of a neuron [32]. Fibers with larger axonal diameter tend to have a higher myelination in order to function optimally. Fibers with large axons usually have an increased energy demand to propagate action potentials [72, 73]. It is thus possible that axons with more myelin, larger diameter and more widely spaced nodal gaps might necessitate a higher metabolic support by oligodendrocytes, depending on their activity [33]. A compelling study in healthy elderly adults observed an increase in cerebral blood flow of the whole brain after complex cognitive training which was subsequently followed by increases in anisotropic diffusion [74]. The authors suggested that the task-related activations of the cortex might have increased myelination, since myelination processes were found to be triggered by neural activity [75, 76]. Moreover, variations of anisotropic diffusion have been associated with inter-individual differences in brain activity, cognitive and motor activity performance [29, 77–79]. Thus, it is conceivable that individual variation in neuronal activity might have contributed to the currently observed findings of a positive association between FA and CBF in the centre of white matter tracts with least well perfused WM across subjects. However, the exact mechanism contributing to a positive association between CBF and anisotropic diffusion across subjects can’t be determined with the current data.

In addition, the current results suggest a positive association between FA and CBF across white matter regions (Fig 4). In 21 subjects out of 39 the association between CBF and FA across white matter regions was significantly positive (see S1 Table). Furthermore, when averaging the tract-specific FA and CBF values across subjects, the association between mean FA and mean CBF values across white matter regions remains positive (Fig 4).

However, the current tract-specific results were at odds with the findings of Aslan and colleagues [11], who demonstrated that tracts with a lower FA tended to have a higher perfusion on average. We suggest that diverging findings might have arisen due to different methodological approaches, such as the correction for PVE, the quantification of CBF, the post-processing of DTI images and the statistical analyses. Although both studies analysed the relationship between white matter CBF and white matter microstructural integrity, they may not be comparable regarding the differing methodological approaches.

Considering the quality of WM perfusion data, a previous study claimed that the measurement of CBF in WM was challenging due to limited image resolution and due to signal loss resulting from long transit times in WM [10]. In white matter, a mean perfusion of 19.7±2.7ml/100g/min across all subjects was observed which was in accordance with previous studies [11, 80]. Van Osch and colleagues further showed that in WM a significant perfusion signal was detected in 70% of voxels after 75 averaged ASL images using background suppressed pseudo-continuous ASL, implemented at 3 Tesla with multislice 2D readout after 1525 milliseconds providing evidence for a reliable detection of ASL signal in WM [15]. In comparison, the current study observed a significant ASL signal in 70% of WM voxels after 140 averaged ASL images and in 84% after 200 averaged ASL images in one subject using pseudo-continuous ASL without background suppression (see S5 Fig). Since differences in the amount of significant ASL signal did depend on the number of acquired volumes within the pCASL frame and the number of acquired volumes differed among subjects, the boxplots in S6 Fig show the proportion of significant perfusion signal for all the subjects as a function of their number of averaged ASL images in GM and in WM (see S6 Fig). Across subjects, 66.5±12.2% of WM voxels showed a significant perfusion signal (see Table 1).

Several potential limiting factors need to be considered. The choice of post-label delay (PLD) time used in the present study should be discussed in detail, as it could influence the values of CBF in WM. The choice of label duration (i.e. 1.72 sec) and the PLD (i.e. 1.1 sec) suggests that signal arriving after 2.82 seconds would be missed. Arrival times in WM are expected to be shorter than this which would not affect the estimation of CBF in WM in this study. However, a shorter than normally recommended PLD could result in greater SNR as signal would be subject to less T1 decay. Moreover, perfusion will be highly dependent on the arrival time of blood: only arrival times less than the PLD will fall in the region of the kinetic curve that would be minimally sensitive to arrival time. For pCASL arrival times exceeding 1 second would be quite normal and even longer in WM [81].

In order to investigate the effect of PLD on ASL signal in white matter, we performed additional ASL measurements in five subjects with three different PLD times (see S16 Fig). We used the same MRI setting for ASL measurement with PLD = 1100 ms, 1525 ms and 1900 ms. We observed that PLD did not significantly affect CBF values within the same white matter regions as presented in Fig 1. We additionally calculated the SNR of ASL signal in the same white matter regions as mentioned above for each PLD (see S17 Fig) and again found no significant effect of the chosen PLD neither on SNR nor on the ASL. Thus, the additional analyses showed that CBF, SNR and variance were not affected substantially by longer PLD keeping all other MRI settings constant (see S16 Fig). Although the recommendations of sequence settings for optimal data acquisition to measure CBF in WM involve higher PLD values than used in the present study [38], we observed ASL signal significantly different from zero and sufficiently high SNR within the significant regions which could not be significantly increased merely by choosing a longer PLD (See S5 and S6 Figs, and S17 Fig). This is in accordance with the findings of Van Osch and colleagues showing that 10 min scanning resulted in significant perfusion signal in 70% of WM voxels, but increasing the labeling and delay time did not lead to a higher percentage [15].

Limitations included the fact that for the quantification of CBF with ASL data, certain standard parameters such as the blood-water partition coefficient, arterial blood relaxation time and labeling efficiency might have been influenced by the tissue type (WM/GM) [48], subjective factors such as scanner stability, brain region, gender and age, sleep wake cycles, psychoactive substances etc. E.g. sex might differentially contribute to partial voluming effects due to the differences in head size between men and women and consequently affect the results. The scatterplot in S13 Fig shows the relationship between CBF and FA in white matter for men and women separately. Although post-hoc analyses showed that gender did not significantly affect the linear regression model between CBF and FA, such nuisance variables were included if possible as covariates in the TBSS analyses (age, sex, number of ASL averages). On the other hand, other nuisance variables were fixed such as the time of MRI to morning hours, or asking the subjects to refrain from psychoactive substances (coffee, alcohol) for at least 12 h before measurement. Since ASL has an inherently lower spatial resolution (~3,6 x 3,6 x 7mm^3^) than DTI (~2 x 2 x 2 mm^3^) and high-resolution T1-weighted data (1 x 1 x 1 mm^3^), partial voluming effects might have occurred in the current study. Thus, partial voluming effects were dealt with in different ways. First, a rigorous estimation of partial voluming with a fast and robust parameter estimation for statistical partial volume models was implemented and used for ASL data (see S1 Fig) [45]. Second, the calibration of CBF was corrected for partial voluming effects. Since grey and white matter have very different kinetics a normal quantification will provide a CBF that is something of a combination of the two tissue types (WM tends to have lower CBF and longer arrival time than GM). With BASIL it was possible to correct the perfusion estimation for the different tissue types using Bayesian inference method for the kinetic model inversion (see Partial Volume Correction) [51]. Third, the statistical analyses were performed in the “core” of WM tracts, since TBSS projects the local highest FA values onto an alignment invariant tract representation (the FA skeleton) [35]. The mean FA skeleton was thresholded at a FA value of 0.3 (see DTI data processing), ensuring that voxels which are primarily grey matter or cerebrospinal fluid are successfully excluded and that the FA skeleton does not extend to the outermost edges of the cortex [35]. Fourth, we corrected for GM partial voluming on a voxel-wise basis in all the statistical analyses carried out with TBSS by entering grey matter masks as individual voxel-wise regressors into the statistical model (see Statistical analysis). A further limitation consists of the assumptions on the basis of anisotropy and the underlying microstructural properties such as myelination and axonal diameter which are difficult to verify within the current DTI parameters. Additional high-resolution and multishell high-angular resolution schemes as well as track-density imaging may provide more information about axonal density, axon diameter distribution and mean axonal diameter [82].

In conclusion, the current study provided evidence that the blood perfusion in white matter is positively related to anisotropic diffusion across subjects. The coupling between WM perfusion and WM microstructure might be explained by individual differences in microstructural properties such as myelination. The physiology of white matter could be of interest in clinical research such as schizophrenia or multiple sclerosis.

## Supporting Information

S1 Fig Segmentation of GM and WM and registration to ASL space. Whole brain (grey scale), grey matter (green) and white matter (blue) masks are shown within the resolution of the T1-weighted image (A, B, C) and after registration to the ASL image (D, E, F, G, H). The masks are shown before (E, F) and after the application of a threshold (G, H). The threshold that was applied to the grey matter mask was 0.4, whereas it was 0.7 for the white matter mask.
(DOCX)

S2 Fig CBF quantification and SNR. The left column shows the axial view of one subject’s brain in the resolution of an ASL image at z = 4 including the respective colour scales. The right column displays the plots for all the values of the x axis of the same brain at z = 4 and y = 74. The first two rows depicts the GM and WM probability maps as extracted after segmentation. Fourth and fifth rows shows cerebral blood flow (CBF) in ml/100g/min for GM and for WM. Six and seventh rows shows the signal to noise ratio (SNR) for the whole slice and for WM. The ability to detect WM perfusion signal in the current ASL data was assessed and presented in the supplementary material according to a previous study (van Osch et al., 2009). On one hand, we probed for the development of the significant perfusion signal in GM and WM as a function of 200 signal averages within a subject. On the other hand each subjects´ significant perfusion signal in WM and GM as a function of the number of signal averages was assessed. Significant perfusion signal was defined as a significantly (*p* < 0.05) different signal from zero for the ASL difference images (control-label) implemented with a non-parametric one-sample t-test in Randomise program of FSL 5.0 (Andersson and Robinson, 2001).
(DOCX)

S3 Fig Motion parameter estimation. The relative voxel displacement root mean square (RMS) in mm (average of rotation and translation parameter differences) is shown as a function of the ASL timeseries. This frame displacement RMS was averaged over the number of subjects (n) within the session.
(DOCX)

S4 Fig Outlier detection in GM CBF. Jittered strip chart of CBF, perfusion variance for GM and WM as well as the ratio of CBF in GM to CBF in WM and the mean relative displacement (MRD) in mm. The 39 subjects that are included in the statistical analyses are displayed in light green. The 4 subjects that were excluded from the statistical analyses because of their low CBF in GM are displayed in light pink.
(DOCX)

S5 Fig ASL signal as a function of the number of averaged CBF images. The percentage of grey (dashed line) and white matter (solid line) voxels that show the presence of a statistically significant signal as a function of ASL averages within one subject. The percentage of voxels that show a statistically significant ASL signal larger than 0 for grey matter and white matter is shown as a function of the number of 200 averaged ASL images within one subject. 80% of GM voxels reach a significant ASL signal after 50 averaged ASL images and 93% of GM voxels after 200 averaged ASL images. WM has a lower percentage of voxels with significant ASL signal with 50% after 50 averaged ASL images, but finally reaches 84% after 200 averaged ASL images.
(DOCX)

S6 Fig Proportion of significant voxels. The percentage of grey matter (left) and white matter (right) voxels that show the presence of a statistically significant signal as a function of number of averaged ASL images. The percentage of voxels with significant ASL signal in relation to the number of ASL signal averages for WM and GM. In 6 subjects 50 timeseries were recorded, in 27 subjects 100, in one subject 150 and in 5 subjects 200 timeseries were acquired. It can be seen that with a higher number of ASL signal averages also the proportion of voxels with a significant ASL signal increases.
(DOCX)

S7 Fig CBF and SNR as a function of ASL averages. This figure shows ASL related metrics in function of the number for ASL images. In the top row, CBF is shown for GM (left) and for WM (right) as a function of the number for ASL images. The bottom row shows the signal to noise ratio for GM (left) and WM (right) as a function of the number for ASL images.
(DOCX)

S8 Fig Plots of subregions of Fig 1. Scatter plots of the mean FA and mean CBF values across subjects within the subregions of the significant positive correlation between CBF and FA across subjects (see Fig 1, Table 2). The subregions were based on John Hopkins University (JHU) WM labels and tracts atlases. Since these atlas labels do not cover the whole region of the observed significant relationship between CBF and FA across subjects, the values might differ slightly from the plot in Fig 1.
(DOCX)

S9 Fig TBSS results uncorrected for multiple comparisons for FA. A) Regions of significant positive correlation between CBF and FA values overlaid on the MNI template at x = 83, y = 109, z = 98 (TFCE p < 0.05). B) The scatterplot displays the mean FA and CBF values, extracted from each subject in the significant regions indicated in red (tbss_fill was used here which “thickened” the TBSS results).
(DOCX)

S10 Fig TBSS results uncorrected for multiple comparisons for RD. A) Regions of significant negative correlation between CBF and RD values overlaid on the MNI template at x = 104, y = 104, z = 84 (TFCE *p* < 0.05). B) The scatterplot displays the mean RD and CBF values, extracted from each subject in the significant regions indicated in red (tbss_fill was used here which “thickened” the TBSS results).
(DOCX)

S11 Fig TBSS results uncorrected for multiple comparisons for MD. A) Regions of significant negative correlation between CBF and MD values overlaid on the MNI template at x = 104, y = 104, z = 84 (TFCE *p* < 0.05). B) The scatterplot displays the mean MD and CBF values, extracted from each subject in the significant regions indicated in red (tbss_fill was used here which “thickened” the TBSS results).
(DOCX)

S12 Fig TBSS results uncorrected for multiple comparisons for AD. A) Regions of significant negative correlation between CBF and AD values overlaid on the MNI template at x = 117, y = 104, z = 99 (TFCE *p* < 0.05). B) The scatterplot displays the mean AD and CBF values, extracted from each subject in the significant regions indicated in red (tbss_fill was used here which “thickened” the TBSS results).
(DOCX)

S13 Fig Relationship between CBF and FA across subjects: effect of gender. Significant positive correlation between mean CBF and FA values (TFCE corrected p < 0.05) as it is shown in Fig 1 shown for women (in red) and men (in blue) separately. A post-hoc analysis of variance of linear model fits showed that the comparison of the two models (model 1 where CBF is modelled by FA values only and model 2 where CBF values are modelled by FA values and gender) did not differ significantly (p = 0.211, F (36, 37) = 1.62).
(DOCX)

S14 Fig SNR signal characteristics. This figure shows the SNR for pure GM, pure WM and for the ASL signal that was extracted for the regions of significant positive correlation between CBF and FA values (regions from Fig 1). The tissue classes were corrected for possible confounding effects originated by partial volume effects.
(DOCX)

S15 Fig Control-label difference. This figure shows the difference values from the subtraction between control and label images for pure GM, pure WM and for the ASL signal that was extracted for the regions of significant positive correlation between CBF and FA values (regions from Fig 1). The tissue classes were corrected for possible confounding effects originated by partial volume effects. These difference values are the input parameters for the CBF quantification ΔM in the equation of page 7.
(DOCX)

S16 Fig CBF values for 3 different postlabel delay values. This figure shows the CBF values for the additional measure on five subjects with three different values of post-label delay time (1100 ms, 1525 ms and 1900 ms). The CBF values in deep WM shows week dependency on PLD demonstrating that the extracted CBF values with short PLD are slightly underestimated as compared to CBF values with higher PLD, however. Testing the linear model that CBF estimation depends on PLD yields the slope not to be different from zero [t(38) = 1.155; p = 0.188] (in WM–PVE corrected) and [t(38) = 1.259; p = 0.174] in corpus callosum.
(DOCX)

S17 Fig SNR values for 3 different postlabel delay values. This figure shows the SNR for the additional measure on five subjects with three different values of post-label delay time (1100 ms, 1525 ms and 1900 ms). The SNR values of WM (PVE) shows no dependency on PLD. Testing the linear model that SNR depends on PLD yields the slope not to be different from zero [t(38) = .487; p = 0.627].
(DOCX)

S1 Table Relationship of CBF and FA across white matter regions for all subjects. Relationship between FA and CBF across fiber tracts for each subject. Pearson’s R with p-value is reported. A positive correlation is observed in all subjects—in 21 from 39 subjects the positive correlation is significant. The WM regions are based on the John Hopkins University (JHU)-ICBM-DTI-81 WM labels atlas (48 labels) and the JHU-WM tractography atlasv (20 tracts) in MNI space [58, 59]. Since the ASL image did not cover the lower parts of the brain, the values which are displayed comprise the following 51 WM regions: the genu of corpus callosum, the body of corpus callosum, the splenium of corpus callosum, the bilateral anterior limb of internal capsule, the bilateral posterior limb of internal capsule, the bilateral retrolenticular part of internal capsule, the bilateral anterior corona radiata, the bilateral superior corona radiata, the bilateral posterior corona radiata, the bilateral posterior thalamic radiation, the bilateral sagittal stratum, the bilateral external capsule, the bilateral cingulum (cingulate gyrus), the bilateral fornix (cres) / Stria terminalis, the bilateral superior longitudinal fasciculus, the bilateral superior fronto-occipital fasciculus, the bilateral tapetum, the bilateral anterior thalamic radiation, the bilateral corticospinal tract, the bilateral cingulum (cingulate gyrus), the bilateral cingulum (hippocampus) the forceps major and minor, the bilateral inferior fronto-occipital fasciculus, the bilateral inferior longitudinal fasciculus, the bilateral superior longitudinal fasciculus, the bilateral uncinate fasciculus, the bilateral superior longitudinal fasciculus (temporal part).
(DOCX)
