# Supplementary material for: Microstructure and Cerebral Blood Flow within White Matter of the Human Brain: A TBSS Analysis
Source: PLoS One. 2016 Mar 4;11(3):e0150657. doi: 10.1371/journal.pone.0150657 (PMC4778945; doi:10.1371/journal.pone.0150657)
Supplement: S1 Fig — Whole brain (grey scale), grey matter (green) and white matter (blue) masks are shown within the resolution of the T1-weighted image (A, B, C) and after registration to the ASL image (D, E, F, G, H). The masks are shown before (E, F) and after the application of a threshold (G, H). The threshold that was applied to the grey matter mask was 0.4, whereas it was 0.7 for the white matter mask. (DOCX) [file pone.0150657.s001.docx]

**Segmentation of GM and WM and registration to ASL space
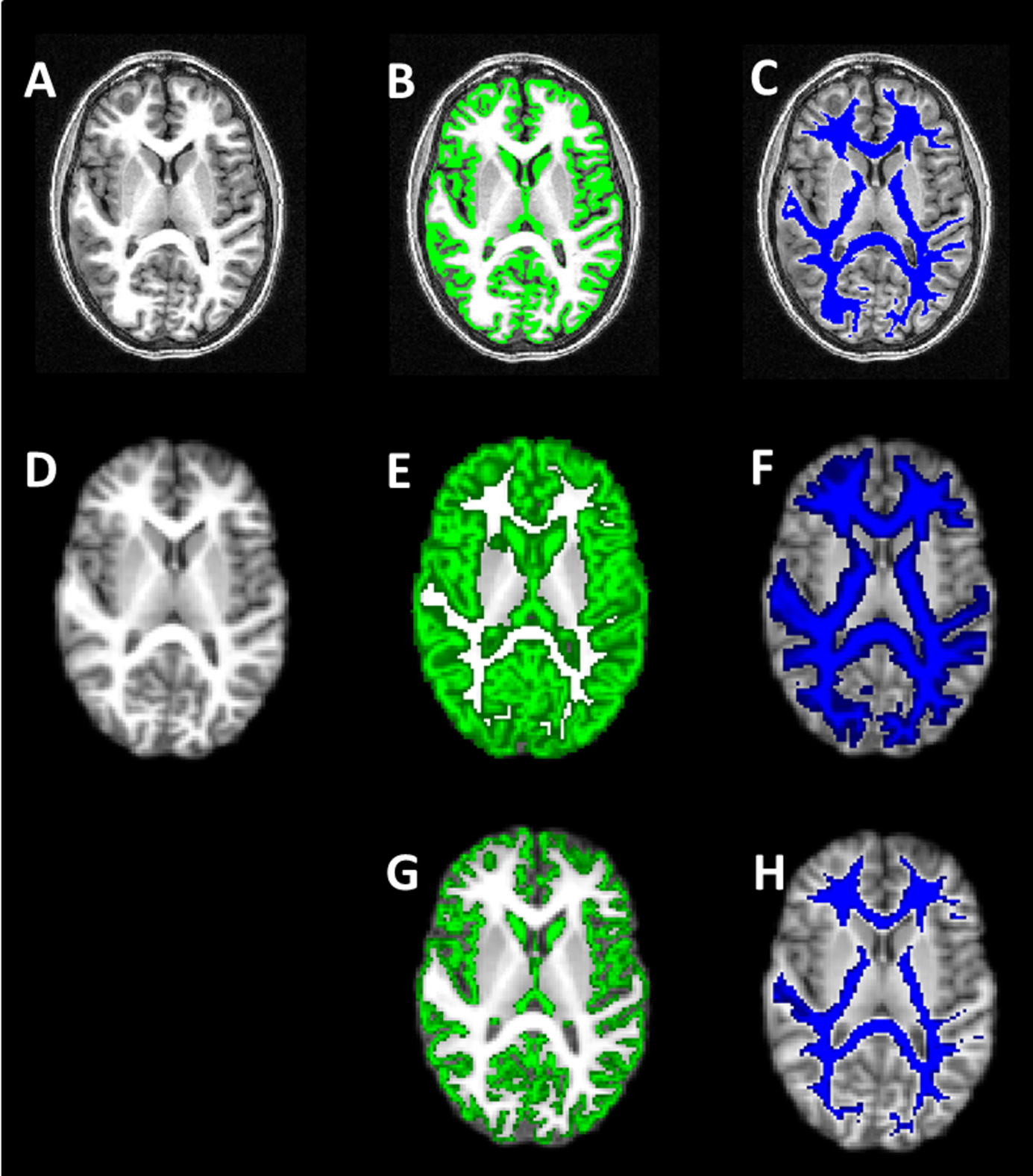
**

**S1 Fig.**

Whole brain (grey scale), grey matter (green) and white matter (blue) masks are shown within the resolution of the T1-weighted image (A, B, C) and after registration to the ASL image (D, E, F, G, H). The masks are shown before (E, F) and after the application of a threshold (G, H). The threshold that was applied to the grey matter mask was 0.4, whereas it was 0.7 for the white matter mask.
