# Supplementary material for: Microstructure and Cerebral Blood Flow within White Matter of the Human Brain: A TBSS Analysis
Source: PLoS One. 2016 Mar 4;11(3):e0150657. doi: 10.1371/journal.pone.0150657 (PMC4778945; doi:10.1371/journal.pone.0150657)
Supplement: S2 Fig — The left column shows the axial view of one subject’s brain in the resolution of an ASL image at z = 4 including the respective colour scales. The right column displays the plots for all the values of the x axis of the same brain at z = 4 and y = 74. The first two rows depicts the GM and WM probability maps as extracted after segmentation. Fourth and fifth rows shows cerebral blood flow (CBF) in ml/100g/min for GM and for WM. Six and seventh rows shows the signal to noise ratio (SNR) for the whole slice and for WM. The ability to detect WM perfusion signal in the current ASL data was assessed and presented in the supplementary material according to a previous study (van Osch et al., 2009). On one hand, we probed for the development of the significant perfusion signal in GM and WM as a function of 200 signal averages within a subject. On the other hand each subjects´ significant perfusion signal in WM and GM as a function of the number of signal averages was assessed. Significant perfusion signal was defined as a significantly (p < 0.05) different signal from zero for the ASL difference images (control-label) implemented with a non-parametric one-sample t-test in Randomise program of FSL 5.0 (Andersson and Robinson, 2001). (DOCX) [file pone.0150657.s002.docx]

**CBF quantification and SNR
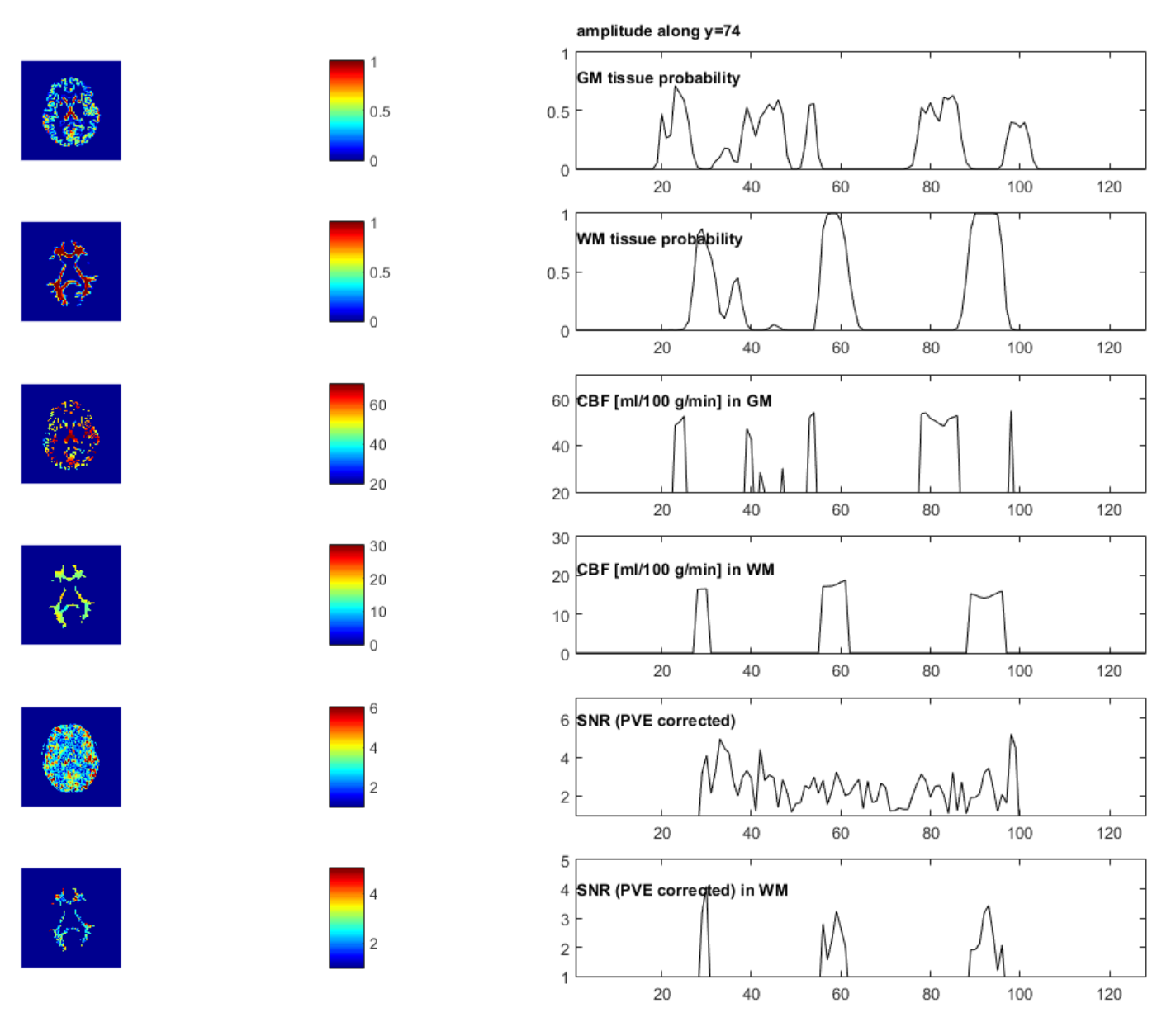
**

**S2 Fig.**

The left column shows the axial view of one subject’s brain in the resolution of an ASL image at z = 4 including the respective colour scales. The right column displays the plots for all the values of the x axis of the same brain at z = 4 and y = 74. The first two rows depicts the GM and WM probability maps as extracted after segmentation. Fourth and fifth rows shows cerebral blood flow (CBF) in ml/100g/min for GM and for WM. Six and seventh rows shows the signal to noise ratio (SNR) for the whole slice and for WM.

The ability to detect WM perfusion signal in the current ASL data was assessed and presented in the supplementary material according to a previous study ([van Osch et al., 2009](#_ENREF_2)). On one hand, we probed for the development of the significant perfusion signal in GM and WM as a function of 200 signal averages within a subject. On the other hand each subjects´ significant perfusion signal in WM and GM as a function of the number of signal averages was assessed. Significant perfusion signal was defined as a significantly (*p* < 0.05) different signal from zero for the ASL difference images (control-label) implemented with a non-parametric one-sample t-test in Randomise program of FSL 5.0 ([Andersson and Robinson, 2001](#_ENREF_1)).
