# Supplementary material for: Microstructure and Cerebral Blood Flow within White Matter of the Human Brain: A TBSS Analysis
Source: PLoS One. 2016 Mar 4;11(3):e0150657. doi: 10.1371/journal.pone.0150657 (PMC4778945; doi:10.1371/journal.pone.0150657)
Supplement: S3 Fig — The relative voxel displacement root mean square (RMS) in mm (average of rotation and translation parameter differences) is shown as a function of the ASL timeseries. This frame displacement RMS was averaged over the number of subjects (n) within the session. (DOCX) [file pone.0150657.s003.docx]

**Motion paramter estimation**


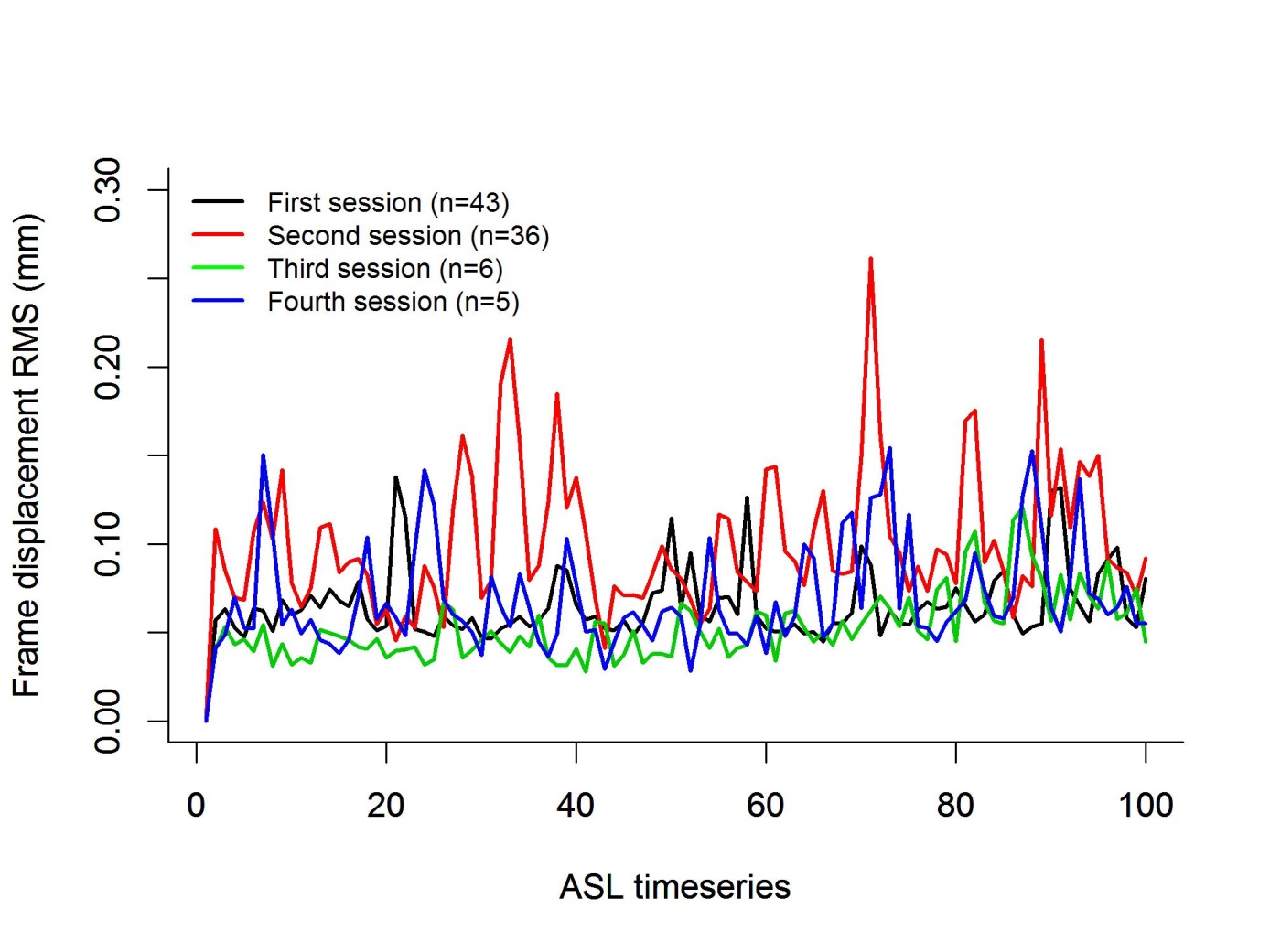


**S3 Fig.**

The relative voxel displacement root mean square (RMS) in mm (average of rotation and translation parameter differences) is shown as a function of the ASL timeseries. This frame displacement RMS was averaged over the number of subjects (n) within the session.
