# Supplementary material for: Microstructure and Cerebral Blood Flow within White Matter of the Human Brain: A TBSS Analysis
Source: PLoS One. 2016 Mar 4;11(3):e0150657. doi: 10.1371/journal.pone.0150657 (PMC4778945; doi:10.1371/journal.pone.0150657)
Supplement: S4 Fig — Jittered strip chart of CBF, perfusion variance for GM and WM as well as the ratio of CBF in GM to CBF in WM and the mean relative displacement (MRD) in mm. The 39 subjects that are included in the statistical analyses are displayed in light green. The 4 subjects that were excluded from the statistical analyses because of their low CBF in GM are displayed in light pink. (DOCX) [file pone.0150657.s004.docx]

**Outlier detection in GM CBF**


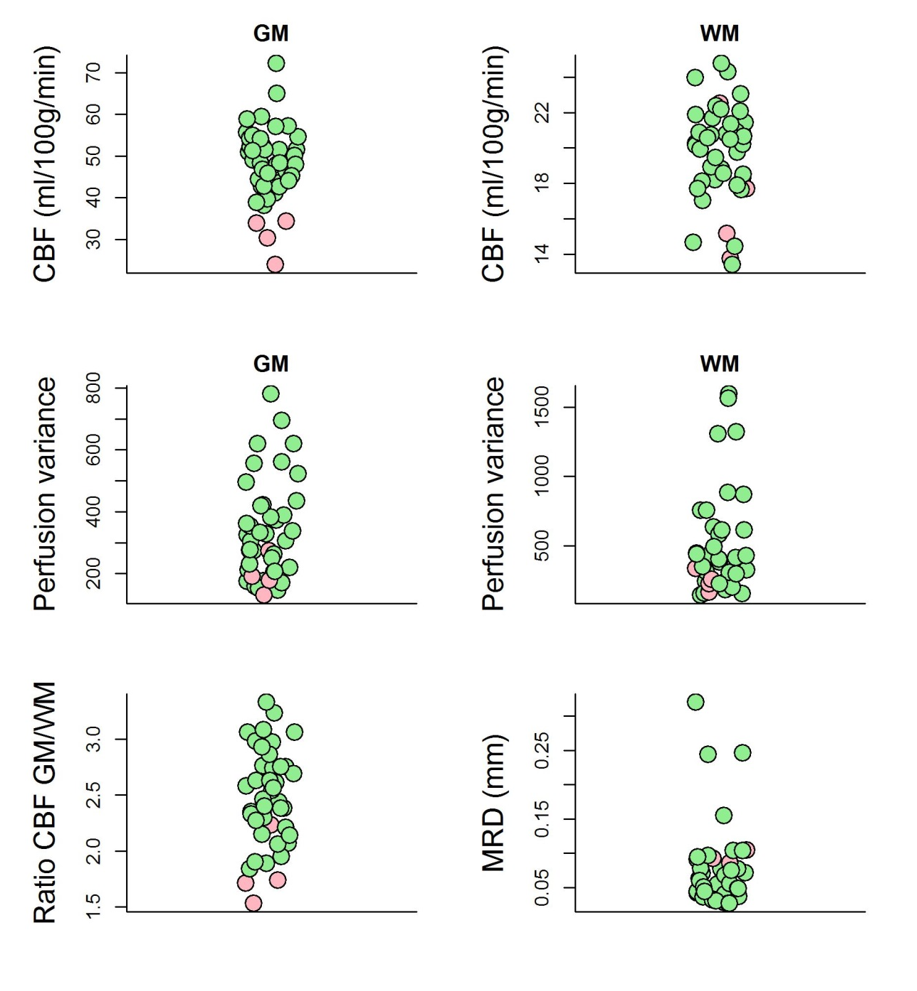


**S4 Fig.**

Jittered strip chart of CBF, perfusion variance for GM and WM as well as the ratio of CBF in GM to CBF in WM and the mean relative displacement (MRD) in mm. The 39 subjects that are included in the statistical analyses are displayed in light green. The 4 subjects that were excluded from the statistical analyses because of their low CBF in GM are displayed in light pink.
