# Supplementary material for: Microstructure and Cerebral Blood Flow within White Matter of the Human Brain: A TBSS Analysis
Source: PLoS One. 2016 Mar 4;11(3):e0150657. doi: 10.1371/journal.pone.0150657 (PMC4778945; doi:10.1371/journal.pone.0150657)
Supplement: S5 Fig — The percentage of grey (dashed line) and white matter (solid line) voxels that show the presence of a statistically significant signal as a function of ASL averages within one subject. The percentage of voxels that show a statistically significant ASL signal larger than 0 for grey matter and white matter is shown as a function of the number of 200 averaged ASL images within one subject. 80% of GM voxels reach a significant ASL signal after 50 averaged ASL images and 93% of GM voxels after 200 averaged ASL images. WM has a lower percentage of voxels with significant ASL signal with 50% after 50 averaged ASL images, but finally reaches 84% after 200 averaged ASL images. (DOCX) [file pone.0150657.s005.docx]

**ASL signal as a function of the number of averaged CBF images**


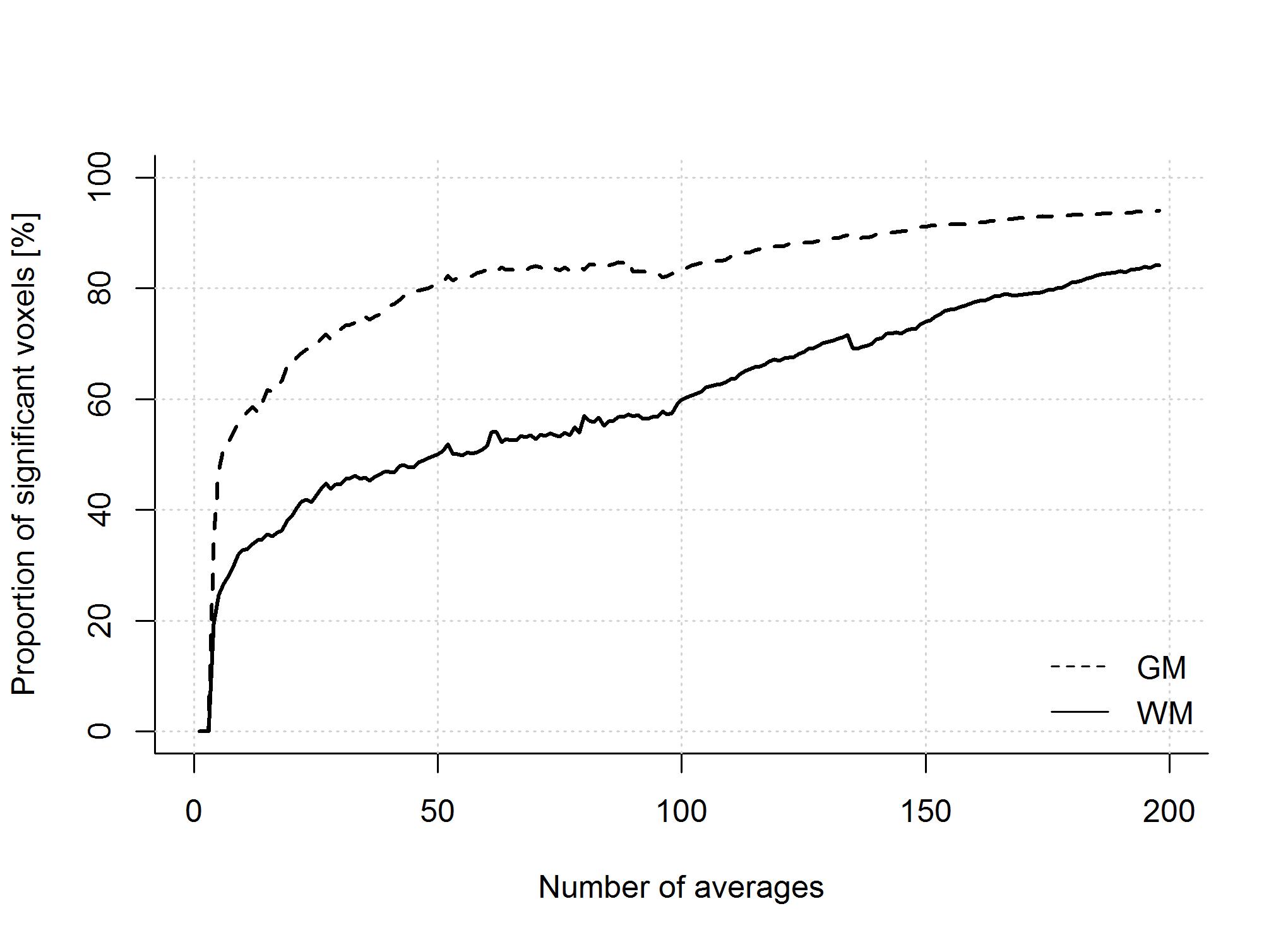


**S5 Fig.**

The percentage of grey (dashed line) and white matter (solid line) voxels that show the presence of a statistically significant signal as a function of ASL averages within one subject.

The percentage of voxels that show a statistically significant ASL signal larger than 0 for grey matter and white matter is shown as a function of the number of 200 averaged ASL images within one subject. 80% of GM voxels reach a significant ASL signal after 50 averaged ASL images and 93% of GM voxels after 200 averaged ASL images. WM has a lower percentage of voxels with significant ASL signal with 50% after 50 averaged ASL images, but finally reaches 84% after 200 averaged ASL images.
