# Supplementary material for: Microstructure and Cerebral Blood Flow within White Matter of the Human Brain: A TBSS Analysis
Source: PLoS One. 2016 Mar 4;11(3):e0150657. doi: 10.1371/journal.pone.0150657 (PMC4778945; doi:10.1371/journal.pone.0150657)
Supplement: S6 Fig — The percentage of grey matter (left) and white matter (right) voxels that show the presence of a statistically significant signal as a function of number of averaged ASL images. The percentage of voxels with significant ASL signal in relation to the number of ASL signal averages for WM and GM. In 6 subjects 50 timeseries were recorded, in 27 subjects 100, in one subject 150 and in 5 subjects 200 timeseries were acquired. It can be seen that with a higher number of ASL signal averages also the proportion of voxels with a significant ASL signal increases. (DOCX) [file pone.0150657.s006.docx]

**Proportion of significant voxels**


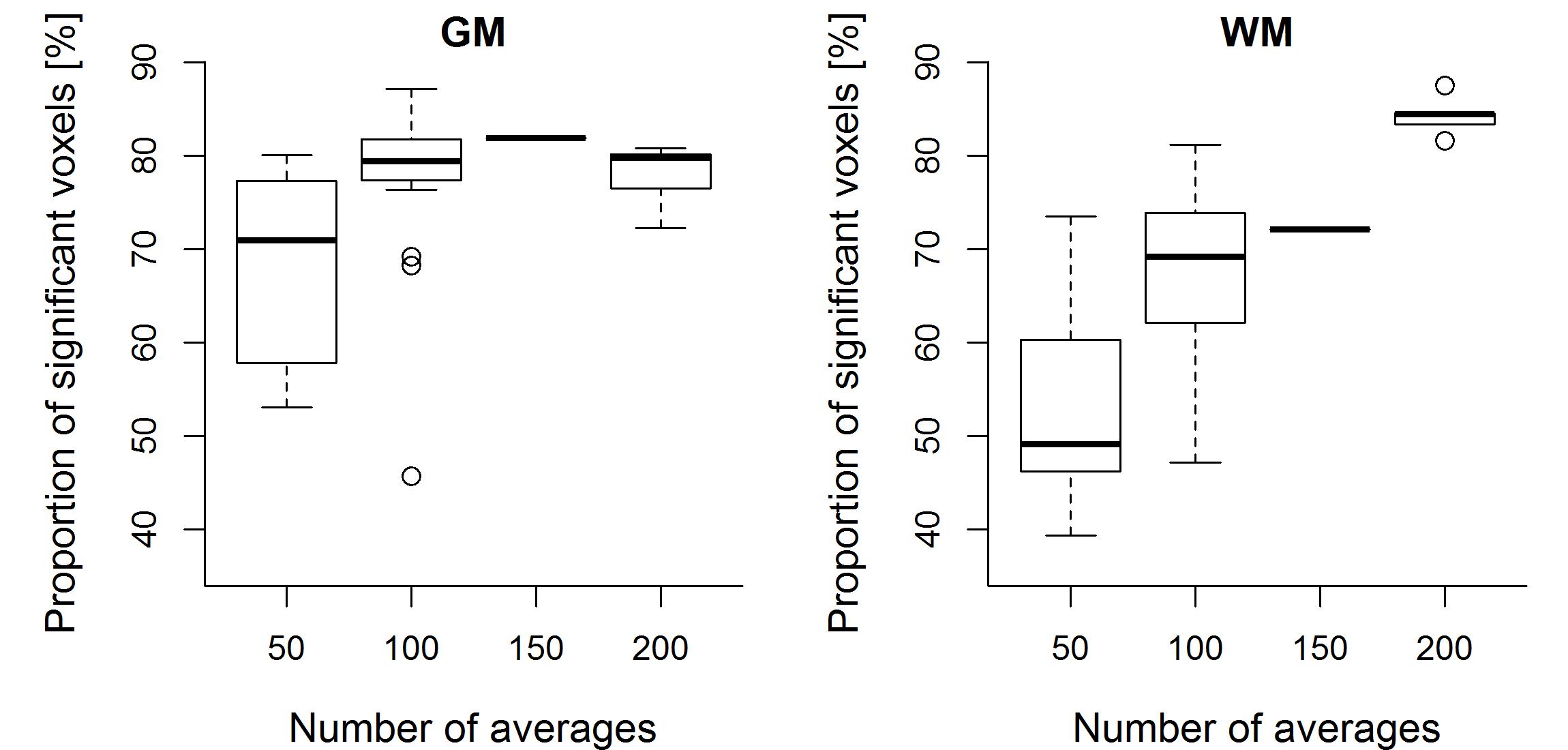


**S6 Fig.**

The percentage of grey matter (left) and white matter (right) voxels that show the presence of a statistically significant signal as a function of number of averaged ASL images.

The percentage of voxels with significant ASL signal in relation to the number of ASL signal averages for WM and GM. In 6 subjects 50 timeseries were recorded, in 27 subjects 100, in one subject 150 and in 5 subjects 200 timeseries were acquired. It can be seen that with a higher number of ASL signal averages also the proportion of voxels with a significant ASL signal increases.
