# Supplementary material for: Microstructure and Cerebral Blood Flow within White Matter of the Human Brain: A TBSS Analysis
Source: PLoS One. 2016 Mar 4;11(3):e0150657. doi: 10.1371/journal.pone.0150657 (PMC4778945; doi:10.1371/journal.pone.0150657)
Supplement: S7 Fig — This figure shows ASL related metrics in function of the number for ASL images. In the top row, CBF is shown for GM (left) and for WM (right) as a function of the number for ASL images. The bottom row shows the signal to noise ratio for GM (left) and WM (right) as a function of the number for ASL images. (DOCX) [file pone.0150657.s007.docx]

**CBF and SNR as a function of ASL averages**


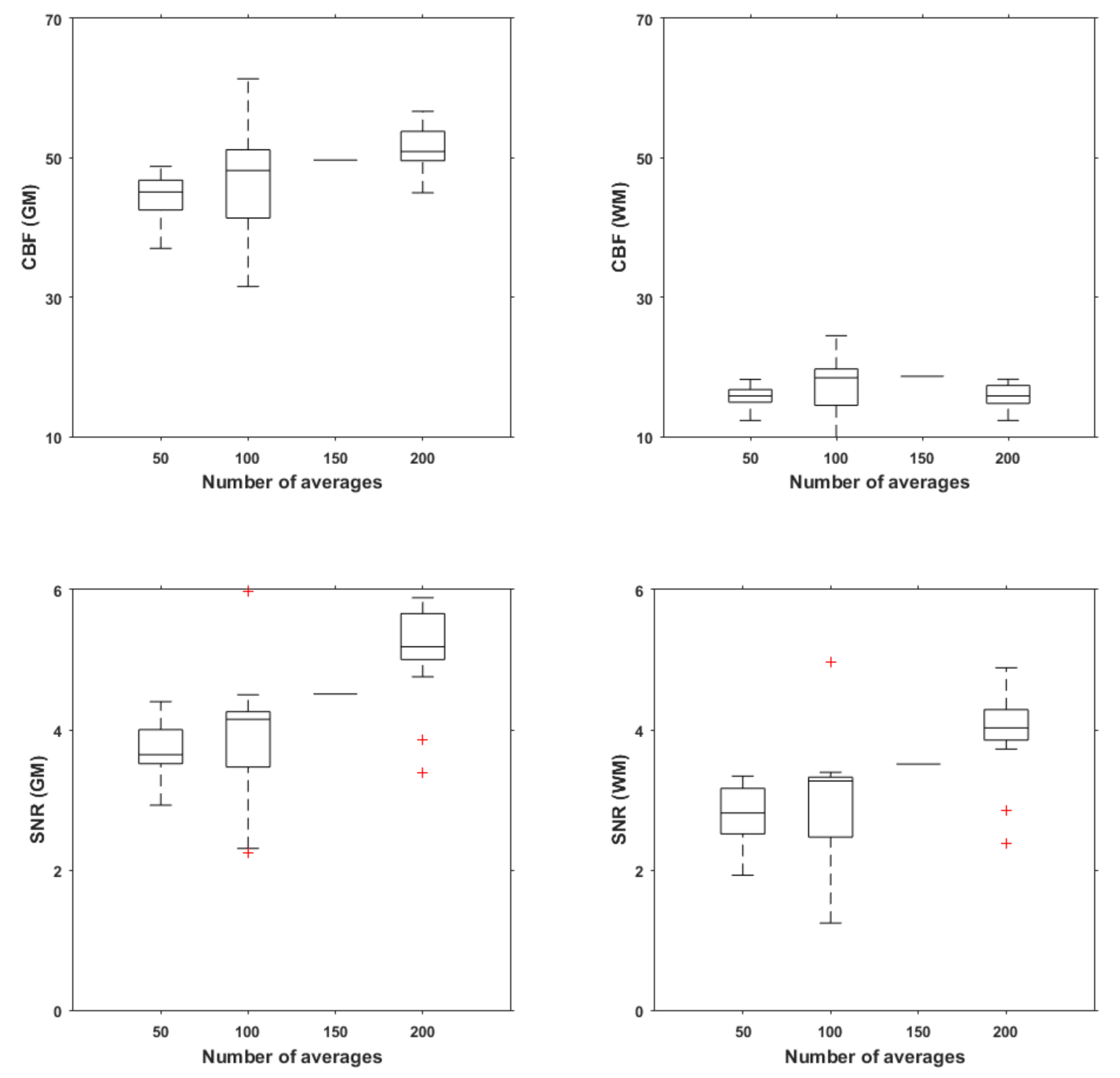


**S7 Fig.**

This figure shows ASL related metrics in function of the number for ASL images. In the top row, CBF is shown for GM (left) and for WM (right) as a function of the number for ASL images. The bottom row shows the signal to noise ratio for GM (left) and WM (right) as a function of the number for ASL images.
