# Supplementary material for: Microstructure and Cerebral Blood Flow within White Matter of the Human Brain: A TBSS Analysis
Source: PLoS One. 2016 Mar 4;11(3):e0150657. doi: 10.1371/journal.pone.0150657 (PMC4778945; doi:10.1371/journal.pone.0150657)
Supplement: S8 Fig — Scatter plots of the mean FA and mean CBF values across subjects within the subregions of the significant positive correlation between CBF and FA across subjects (see Fig 1, Table 2). The subregions were based on John Hopkins University (JHU) WM labels and tracts atlases. Since these atlas labels do not cover the whole region of the observed significant relationship between CBF and FA across subjects, the values might differ slightly from the plot in Fig 1. (DOCX) [file pone.0150657.s008.docx]

**Plots of subregions of Figure 1**


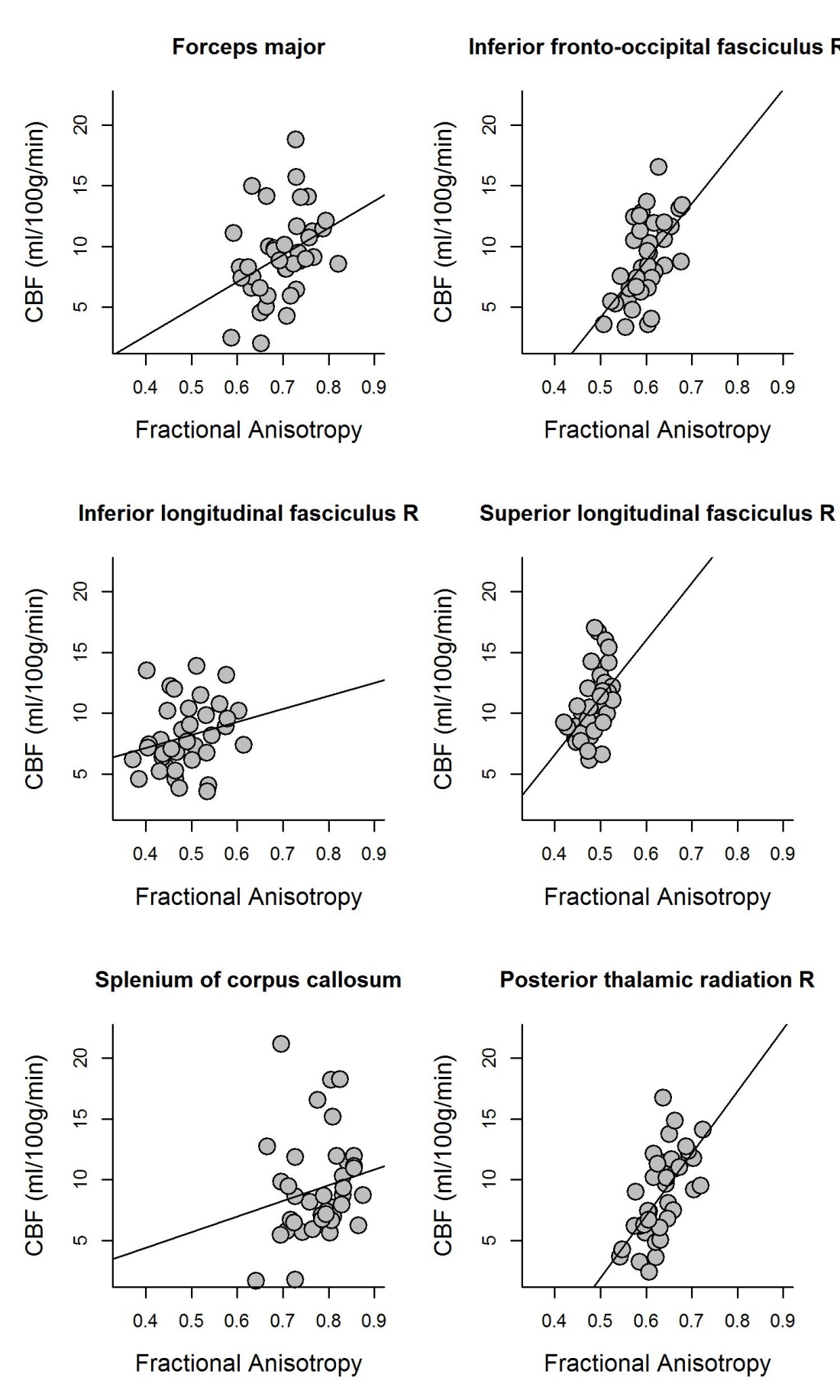


**S8 Fig.**

Scatter plots of the mean FA and mean CBF values across subjects within the subregions of the significant positive correlation between CBF and FA across subjects (see Fig 1, Table 2). The subregions were based on John Hopkins University (JHU) WM labels and tracts atlases. Since these atlas labels do not cover the whole region of the observed significant relationship between CBF and FA across subjects, the values might differ slightly from the plot in Fig 1.
