# Supplementary material for: Microstructure and Cerebral Blood Flow within White Matter of the Human Brain: A TBSS Analysis
Source: PLoS One. 2016 Mar 4;11(3):e0150657. doi: 10.1371/journal.pone.0150657 (PMC4778945; doi:10.1371/journal.pone.0150657)
Supplement: S10 Fig — A) Regions of significant negative correlation between CBF and RD values overlaid on the MNI template at x = 104, y = 104, z = 84 (TFCE p < 0.05). B) The scatterplot displays the mean RD and CBF values, extracted from each subject in the significant regions indicated in red (tbss_fill was used here which “thickened” the TBSS results). (DOCX) [file pone.0150657.s010.docx]

**TBSS results uncorrected for multiple comparisons for RD**
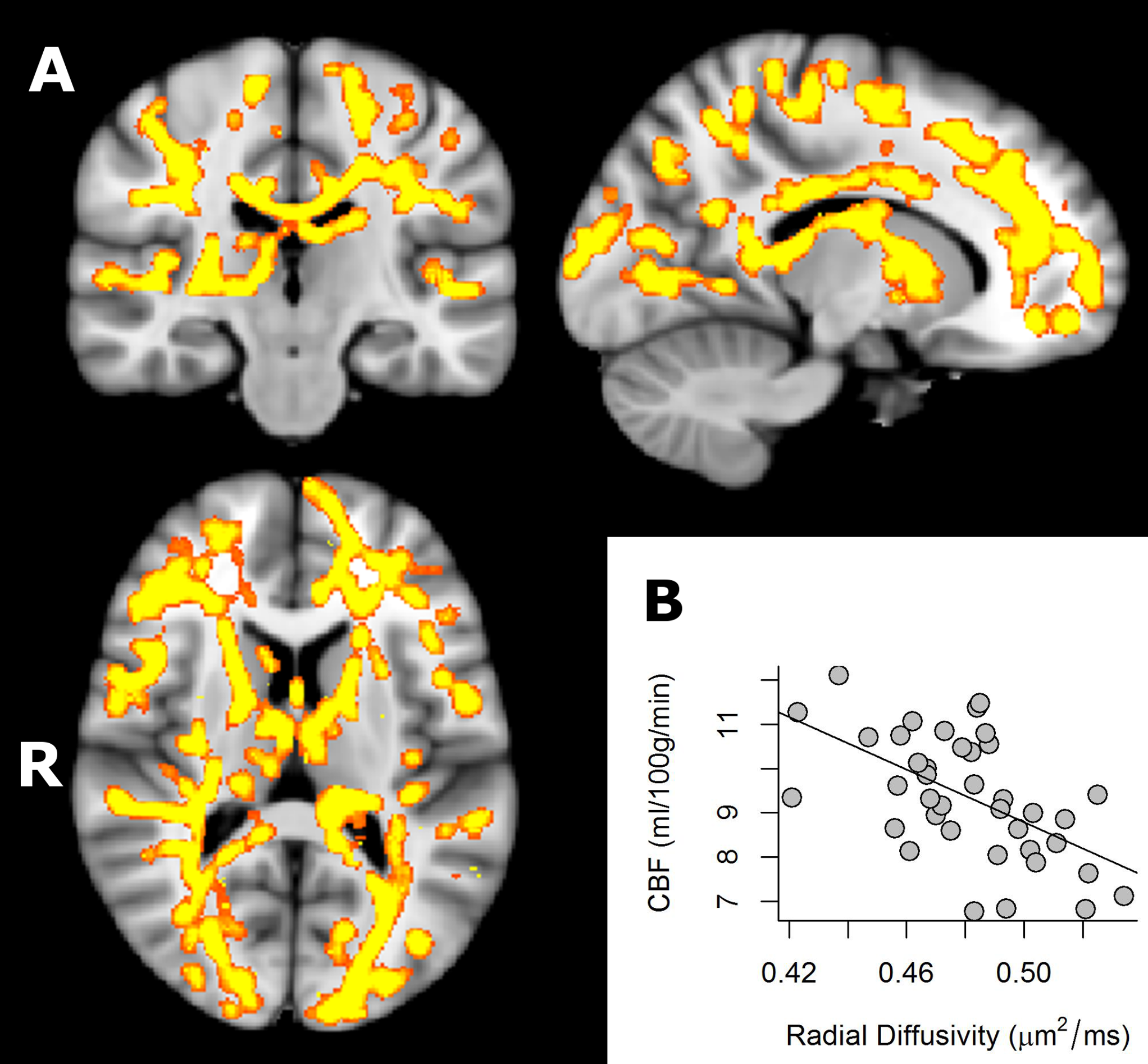


**S10 Fig.**

A) Regions of significant negative correlation between CBF and RD values overlaid on the MNI template at x = 104, y = 104, z = 84 (TFCE *p* < 0.05). B) The scatterplot displays the mean RD and CBF values, extracted from each subject in the significant regions indicated in red (tbss_fill was used here which “thickened” the TBSS results).


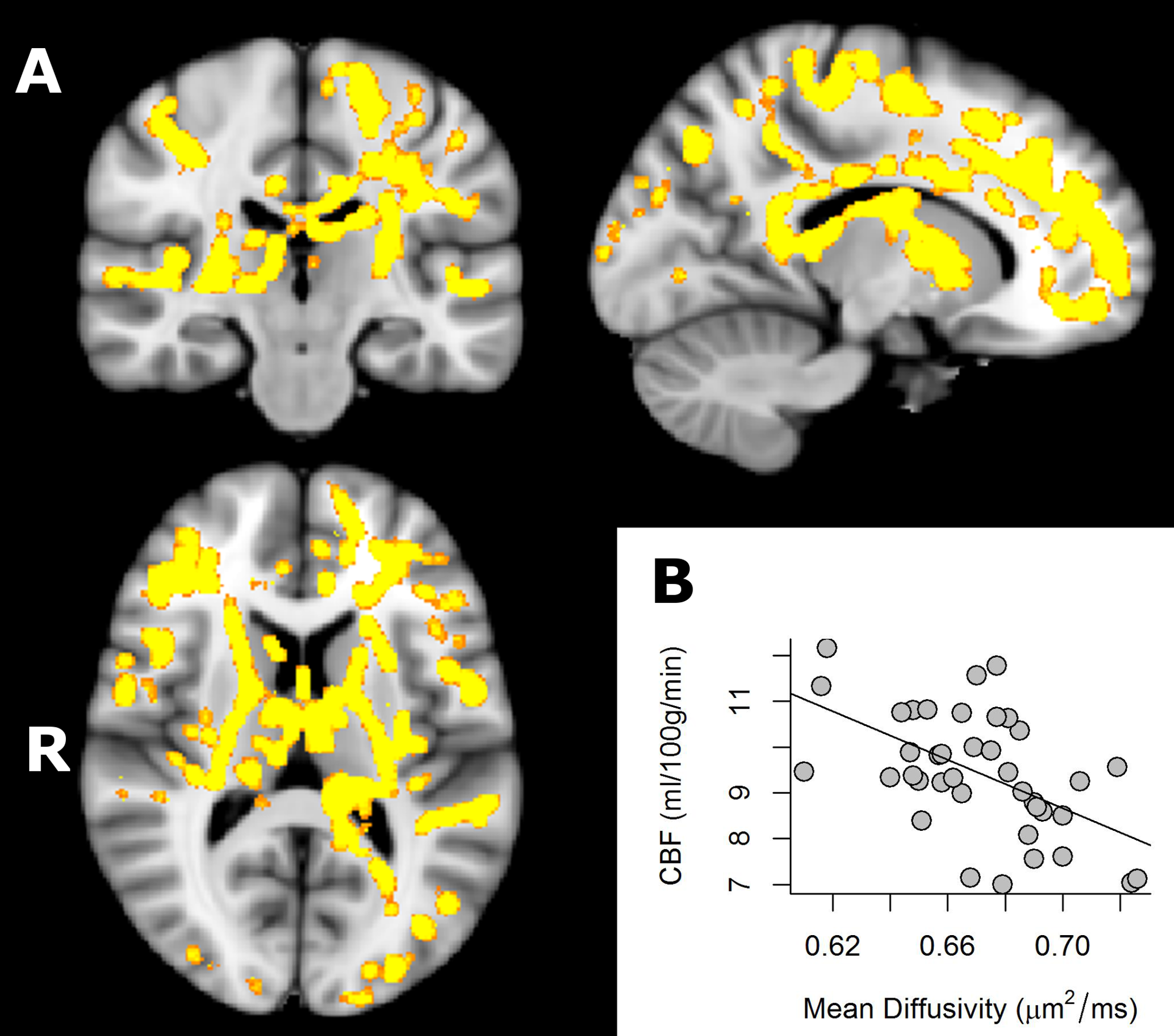


**Figure S11.**

A) Regions of significant negative correlation between CBF and MD values overlaid on the MNI template at x = 104, y = 104, z = 84 (TFCE *p* < 0.05). B) The scatterplot displays the mean MD and CBF values, extracted from each subject in the significant regions indicated in red (tbss_fill was used here which “thickened” the TBSS results).


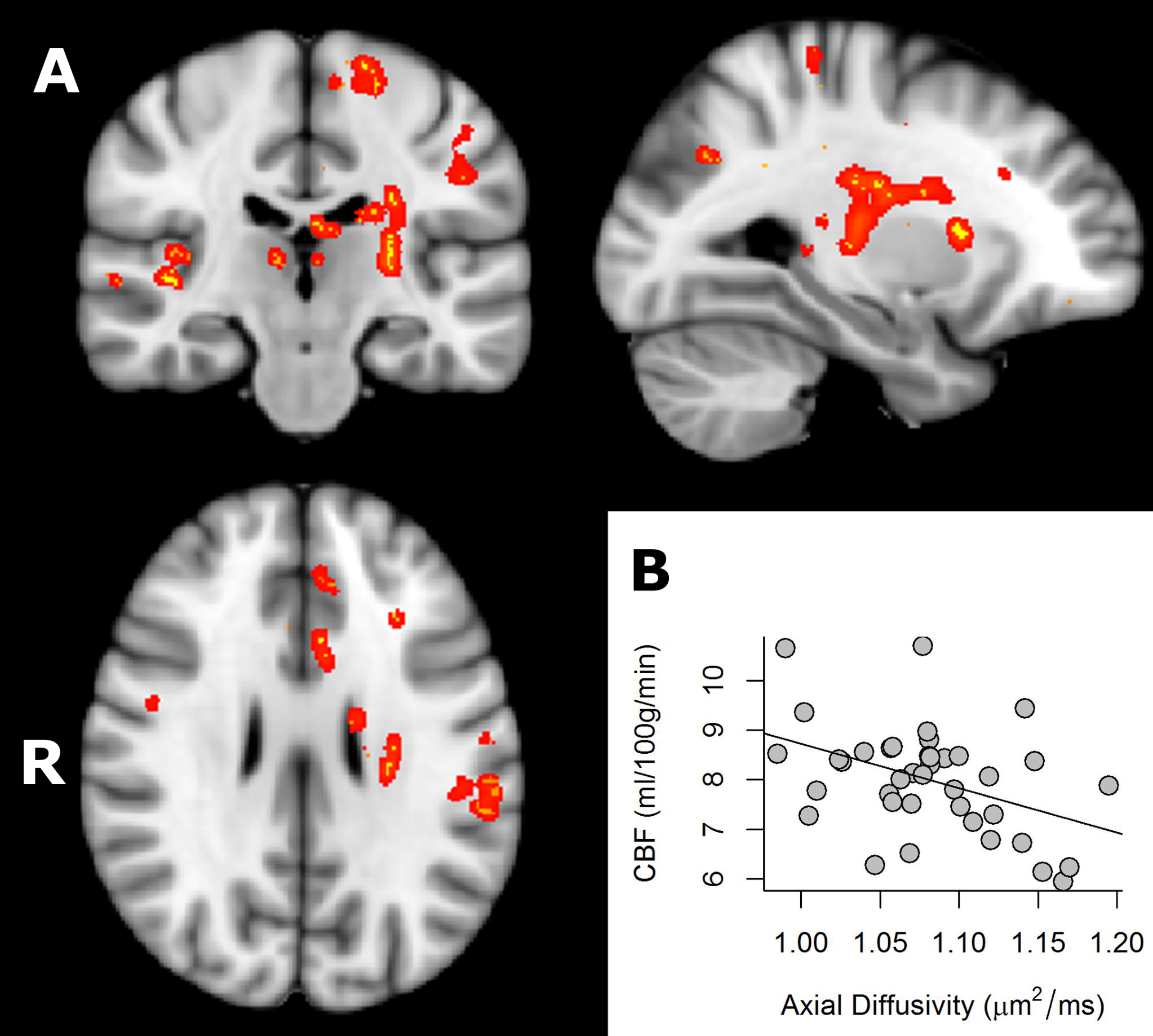


**Figure S12.**

A) Regions of significant negative correlation between CBF and AD values overlaid on the MNI template at x = 117, y = 104, z = 99 (TFCE *p* < 0.05). B) The scatterplot displays the mean AD and CBF values, extracted from each subject in the significant regions indicated in red (tbss_fill was used here which “thickened” the TBSS results).

- 1. **Relationship between CBF and FA across subjects: effect of gender**

**
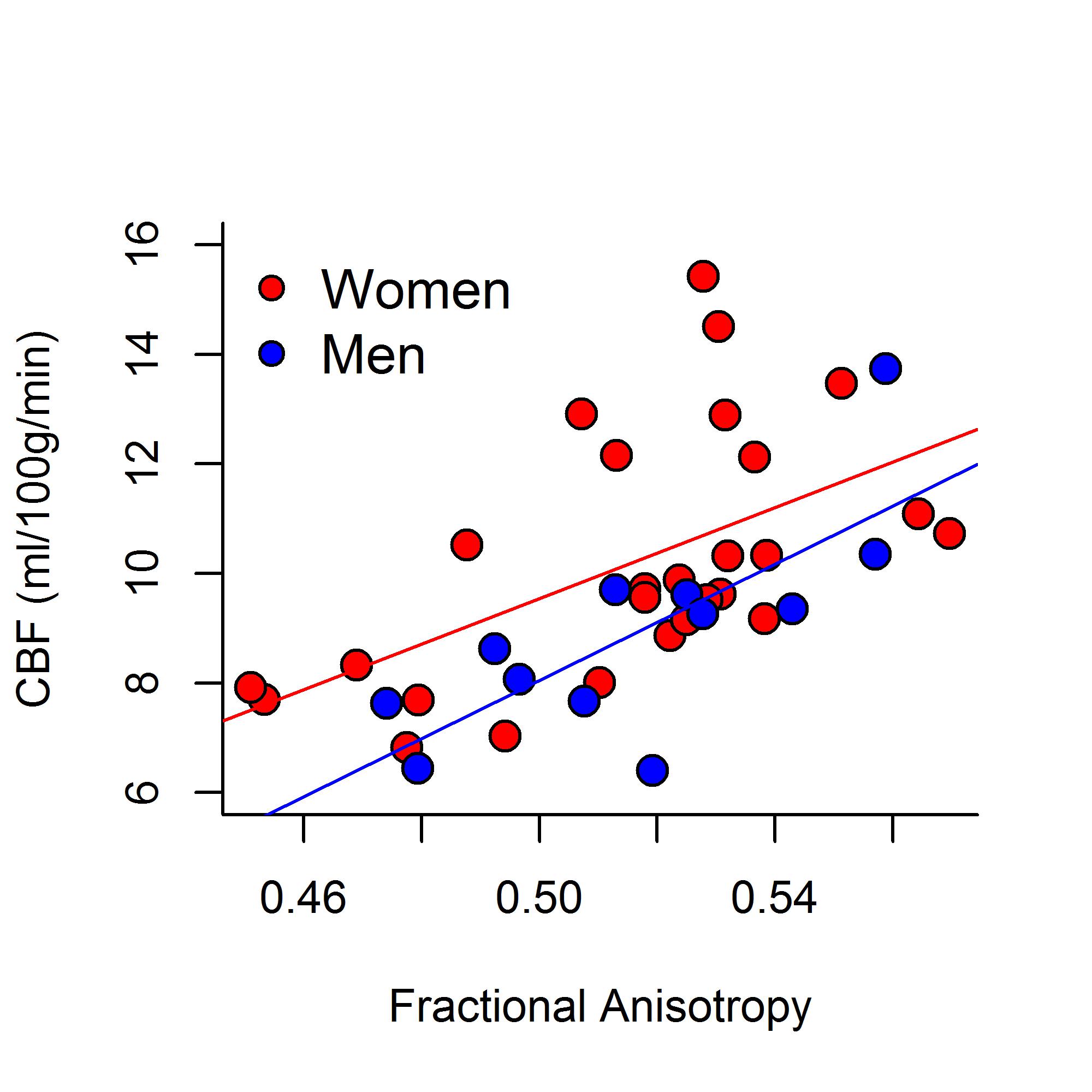
**

**Figure S13**

Significant positive correlation between mean CBF and FA values (TFCE corrected p < 0.05) as it is shown in Figure 1 shown for women (in red) and men (in blue) separately. A post-hoc analysis of variance of linear model fits showed that the comparison of the two models (model 1 where CBF is modelled by FA values only and model 2 where CBF values are modelled by FA values and gender) did not differ significantly (p = 0.211, F (36, 37) = 1.62).

- 1. **Relationship of CBF and FA across white matter regions for all subjects**

| Subject | dRdPearson’s r | p-value |
| --- | --- | --- |
| S1 * | 0.323 | 0.017 |
| S2 | 0.238 | 0.083 |
| S3 * | 0.382 | 0.005 |
| S4 | 0.258 | 0.062 |
| S5 * | 0.407 | 0.003 |
| S6 | 0.208 | 0.135 |
| S7 * | 0.33 | 0.015 |
| S8 | 0.195 | 0.162 |
| S9 * | 0.294 | 0.031 |
| S10 * | 0.341 | 0.013 |
| S11 | 0.17 | 0.22 |
| S12 * | 0.319 | 0.019 |
| S13 | 0.058 | 0.679 |
| S14 | 0.215 | 0.118 |
| S15 * | 0.37 | 0.006 |
| S16 | 0.109 | 0.433 |
| S17 | 0.146 | 0.293 |
| S18 * | 0.268 | 0.05 |
| S19 * | 0.278 | 0.042 |
| S20 * | 0.376 | 0.005 |
| S21 * | 0.326 | 0.017 |
| S22 * | 0.274 | 0.047 |
| S23 | 0.125 | 0.366 |
| S24 * | 0.318 | 0.019 |
| S25 * | 0.325 | 0.016 |
| S26 * | 0.299 | 0.03 |
| S27 | 0.18 | 0.193 |
| S28 | 0.092 | 0.506 |
| S29 * | 0.549 | <0.001 |
| S30 * | 0.297 | 0.029 |
| S31 * | 0.029 | 0.048 |
| S32 | 0.23 | 0.835 |
| S33 | 0.177 | 0.098 |
| S34 | 0.067 | 0.201 |
| S35 | 0.29 | 0.631 |
| S36 * | 0.387 | 0.035 |
| S37 * | 0.057 | 0.004 |
| S38 | 0.323 | 0.68 |
| S39 | 0.198 | 0.152 |

**Table S1.**

Relationship between FA and CBF across fiber tracts for each subject. Pearson’s R with p-value is reported. A positive correlation is observed in all subjects - in 21 from 39 subjects the positive correlation is significant. The WM regions are based on the John Hopkins University (JHU)-ICBM-DTI-81 WM labels atlas (48 labels) and the JHU-WM tractography atlasv (20 tracts) in MNI space ([Mazziotta et al., 2001](#_ENREF_1); [Mori et al., 2005](#_ENREF_2)). Since the ASL image did not cover the lower parts of the brain, the values which are displayed comprise the following 51 WM regions: the genu of corpus callosum, the body of corpus callosum, the splenium of corpus callosum, the bilateral anterior limb of internal capsule, the bilateral posterior limb of internal capsule, the bilateral retrolenticular part of internal capsule, the bilateral anterior corona radiata, the bilateral superior corona radiata, the bilateral posterior corona radiata, the bilateral posterior thalamic radiation, the bilateral sagittal stratum, the bilateral external capsule, the bilateral cingulum (cingulate gyrus), the bilateral fornix (cres) / Stria terminalis, the bilateral superior longitudinal fasciculus, the bilateral superior fronto-occipital fasciculus, the bilateral tapetum, the bilateral anterior thalamic radiation, the bilateral corticospinal tract, the bilateral cingulum (cingulate gyrus), the bilateral cingulum (hippocampus) the forceps major and minor, the bilateral inferior fronto-occipital fasciculus, the bilateral inferior longitudinal fasciculus, the bilateral superior longitudinal fasciculus, the bilateral uncinate fasciculus, the bilateral superior longitudinal fasciculus (temporal part).

**
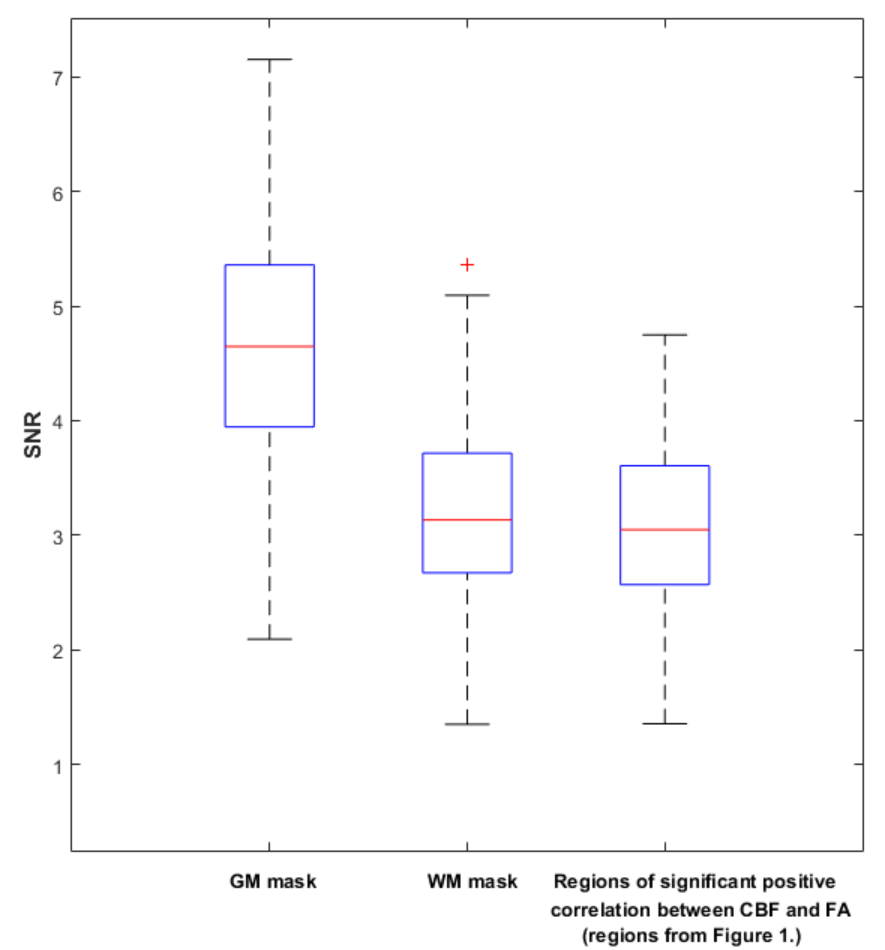
**

**Figure S14.**

This figure shows the SNR for pure GM, pure WM and for the ASL signal that was extracted for the regions of significant positive correlation between CBF and FA values (regions from Figure 1). The tissue classes were corrected for possible confounding effects originated by partial volume effects.


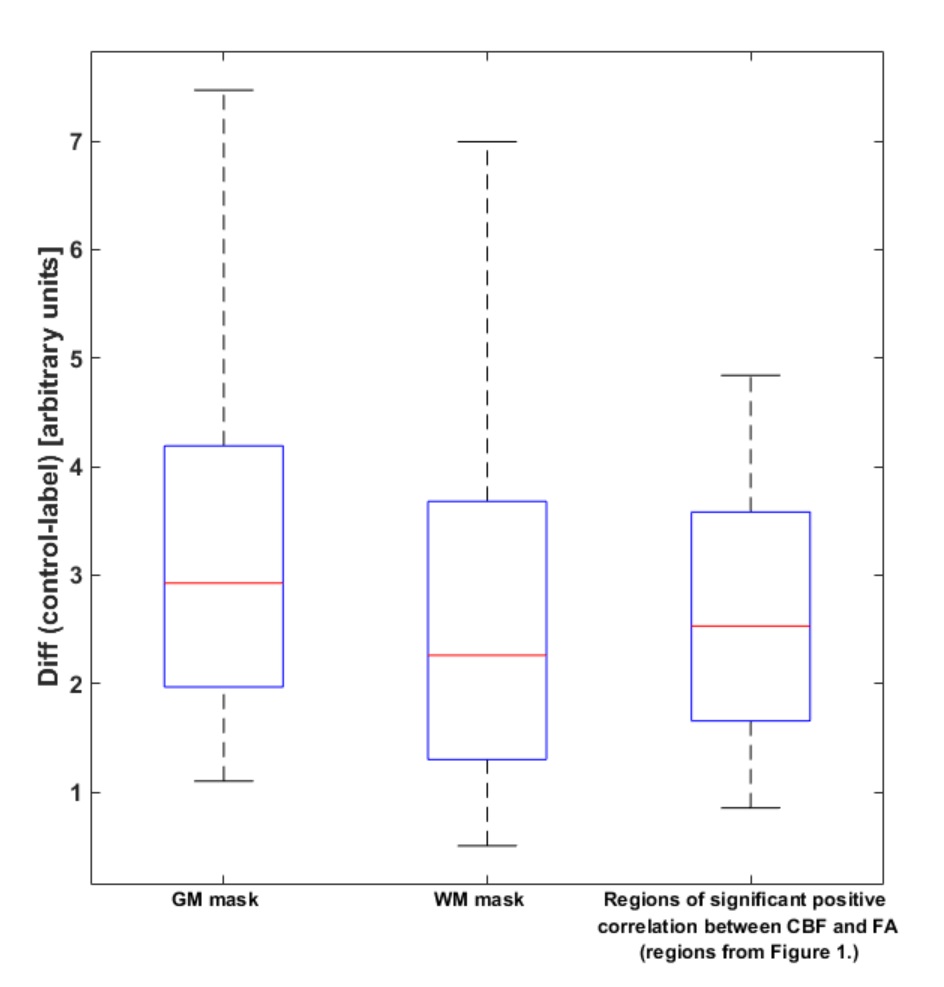


**Figure S15.**

This figure shows the difference values from the subtraction between control and label images for pure GM, pure WM and for the ASL signal that was extracted for the regions of significant positive correlation between CBF and FA values (regions from Figure 1). The tissue classes were corrected for possible confounding effects originated by partial volume effects. These difference values are the input parameters for the CBF quantification ΔM in the equation of page 7.

**
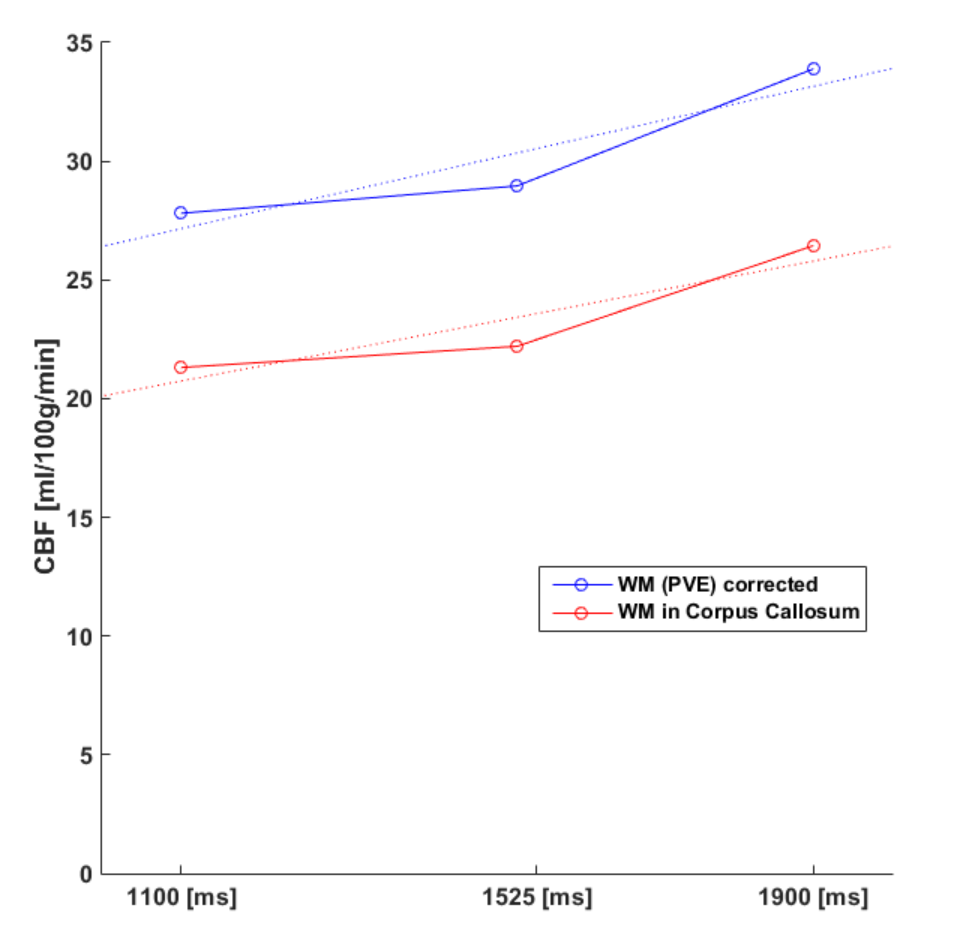
**

**Figure S16.**

This figure shows the CBF values for the additional measure on five subjects with three different values of post-label delay time (1100 ms, 1525 ms and 1900 ms). The CBF values in deep WM shows week dependency on PLD demonstrating that the extracted CBF values with short PLD are slightly underestimated as compared to CBF values with higher PLD, however. Testing the linear model that CBF estimation depends on PLD yields the slope not to be different from zero [t(38)=1.155; p=0.188] (in WM–PVE corrected) and [t(38)=1.259; p=0.174] in corpus callosum.

**
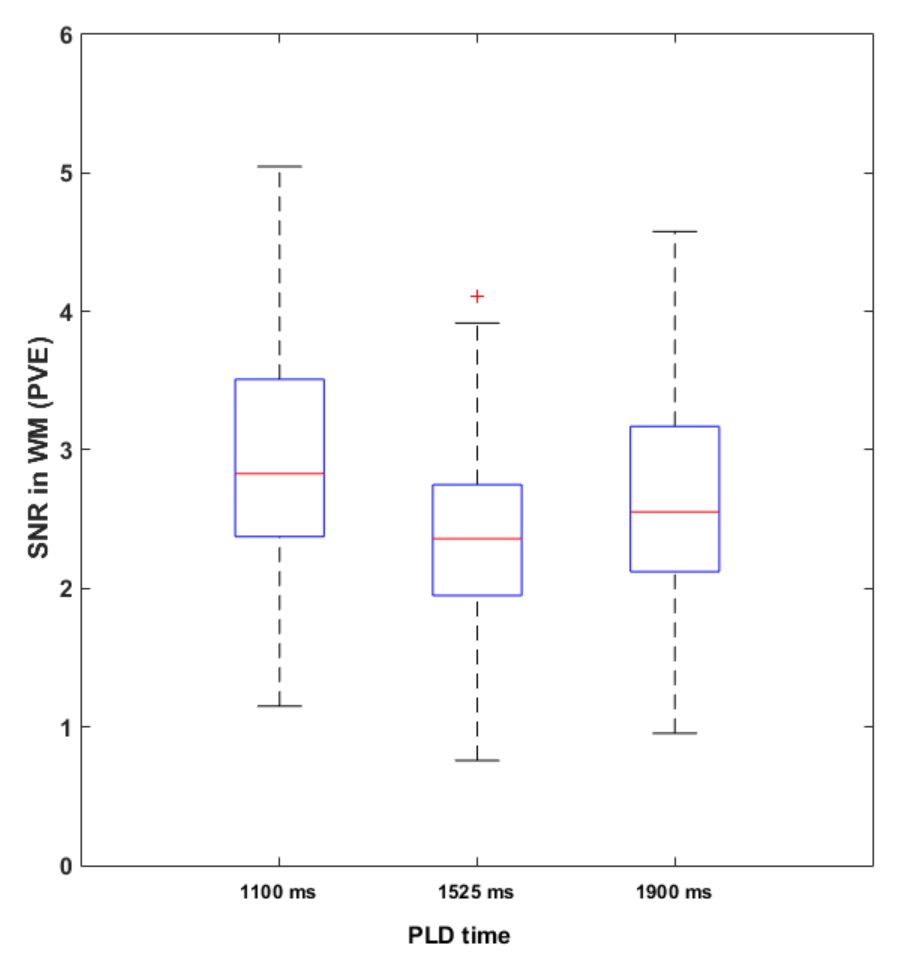
**

**Figure S17.**

This figure shows the SNR for the additional measure on five subjects with three different values of post-label delay time (1100 ms, 1525 ms and 1900 ms). The SNR values of WM (PVE) shows no dependency on PLD. Testing the linear model that SNR depends on PLD yields the slope not to be different from zero [t(38)=.487; p=0.627].

1. **References**

Mazziotta, J.C., Toga, A.W., Evans, A., Fox, P., Lancaster, J., 2001. A probabilistic atlas and reference system for the human brain: International Consortium for Brain Mapping (ICBM). Phil. Trans. R. Soc. Lond. B 356, 1293-1322.

Mori, S., Wakana, S., Nagae-Poetscher, L.M., Van Zijl, P.C.M., 2005. MRI Atlas of Human White Matter. Elsevier, Amsterdam.

Andersson, M.J., Robinson, J., 2001. Permutation Tests for Linear Models. Aust. N. Z. J. Stat. 43, 75-88.

Mazziotta, J.C., Toga, A.W., Evans, A., Fox, P., Lancaster, J., 2001. A probabilistic atlas and reference system for the human brain: International Consortium for Brain Mapping (ICBM). Phil. Trans. R. Soc. Lond. B 356, 1293-1322.

Mori, S., Wakana, S., Nagae-Poetscher, L.M., Van Zijl, P.C.M., 2005. MRI Atlas of Human White Matter. Elsevier, Amsterdam.

van Osch, M.J., Teeuwisse, W.M., van Walderveen, M.A., Hendrikse, J., Kies, D.A., van Buchem, M.A., 2009. Can arterial spin labeling detect white matter perfusion signal? Magn Reson Med 62, 165-173.

Andersson, M.J., Robinson, J., 2001. Permutation Tests for Linear Models. Aust. N. Z. J. Stat. 43, 75-88.

van Osch, M.J., Teeuwisse, W.M., van Walderveen, M.A., Hendrikse, J., Kies, D.A., van Buchem, M.A., 2009. Can arterial spin labeling detect white matter perfusion signal? Magn Reson Med 62, 165-173
