# Supplementary material for: Microstructure and Cerebral Blood Flow within White Matter of the Human Brain: A TBSS Analysis
Source: PLoS One. 2016 Mar 4;11(3):e0150657. doi: 10.1371/journal.pone.0150657 (PMC4778945; doi:10.1371/journal.pone.0150657)
Supplement: S12 Fig — A) Regions of significant negative correlation between CBF and AD values overlaid on the MNI template at x = 117, y = 104, z = 99 (TFCE p < 0.05). B) The scatterplot displays the mean AD and CBF values, extracted from each subject in the significant regions indicated in red (tbss_fill was used here which “thickened” the TBSS results). (DOCX) [file pone.0150657.s012.docx]

**TBSS results uncorrected for multiple comparisons for AD**
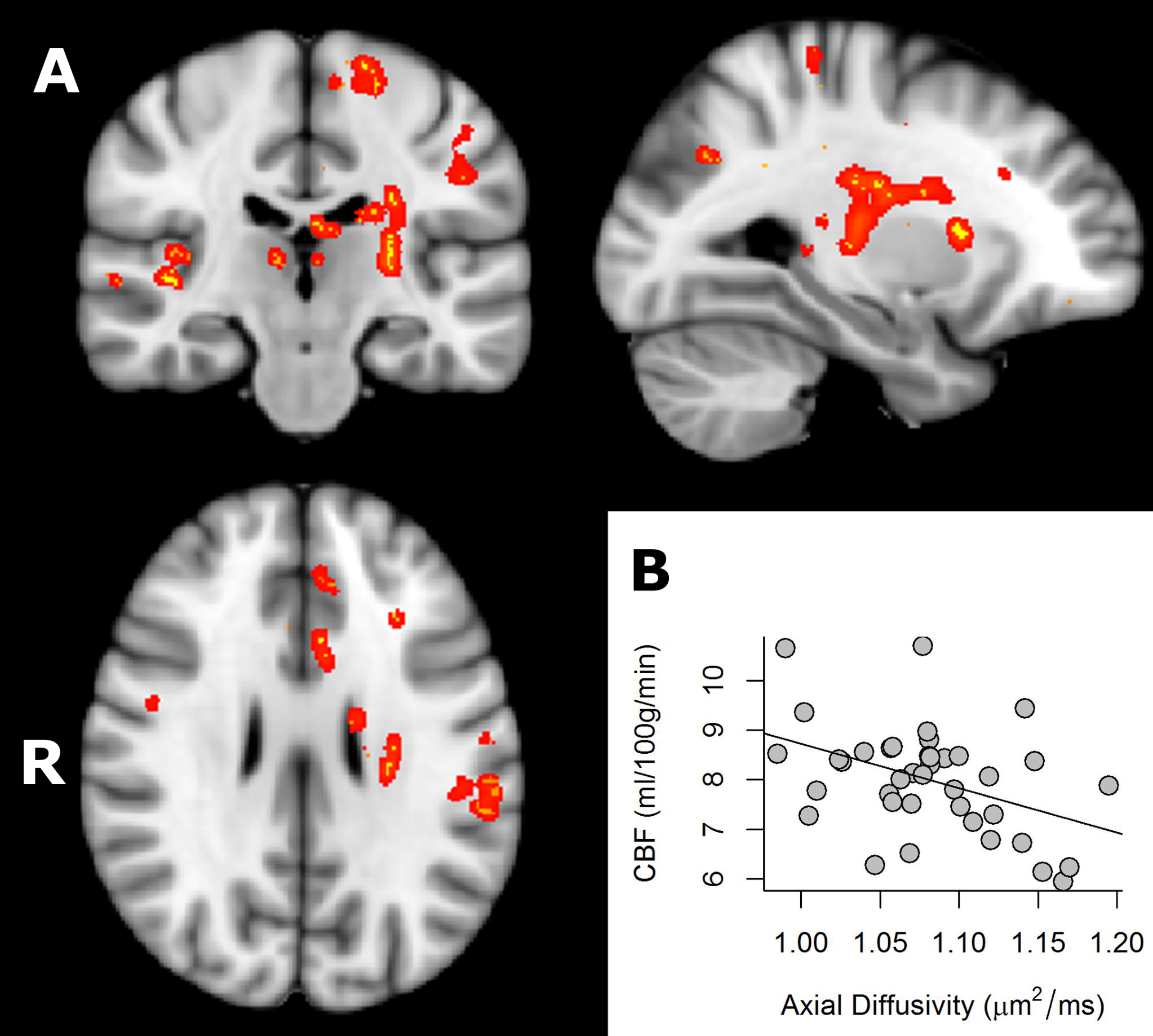


**S12 Fig.**

A) Regions of significant negative correlation between CBF and AD values overlaid on the MNI template at x = 117, y = 104, z = 99 (TFCE *p* < 0.05). B) The scatterplot displays the mean AD and CBF values, extracted from each subject in the significant regions indicated in red (tbss_fill was used here which “thickened” the TBSS results).
