# Supplementary material for: Microstructure and Cerebral Blood Flow within White Matter of the Human Brain: A TBSS Analysis
Source: PLoS One. 2016 Mar 4;11(3):e0150657. doi: 10.1371/journal.pone.0150657 (PMC4778945; doi:10.1371/journal.pone.0150657)
Supplement: S13 Fig — Significant positive correlation between mean CBF and FA values (TFCE corrected p < 0.05) as it is shown in Fig 1 shown for women (in red) and men (in blue) separately. A post-hoc analysis of variance of linear model fits showed that the comparison of the two models (model 1 where CBF is modelled by FA values only and model 2 where CBF values are modelled by FA values and gender) did not differ significantly (p = 0.211, F (36, 37) = 1.62). (DOCX) [file pone.0150657.s013.docx]

**Relationship between CBF and FA across subjects: effect of gender**

**
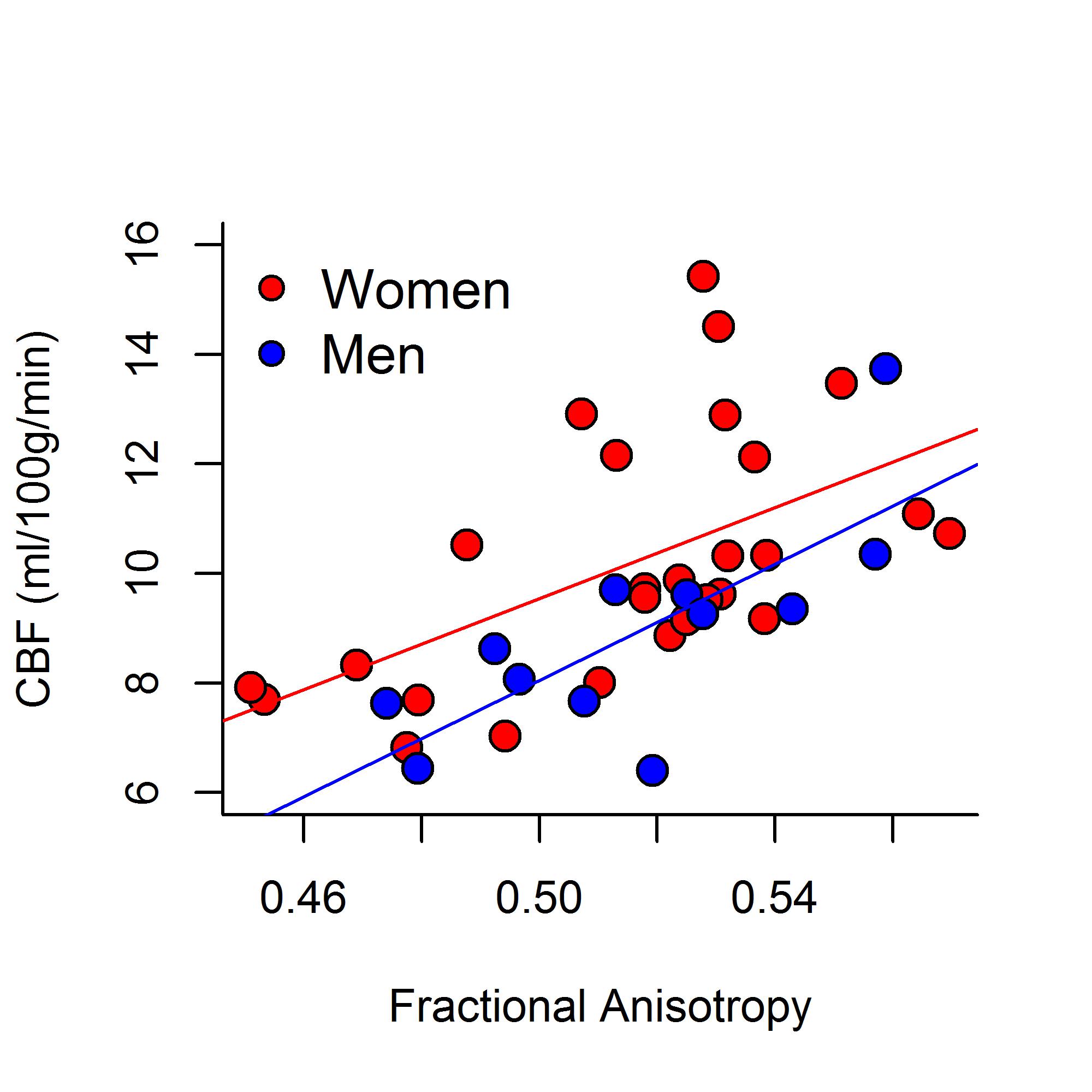
**

**S13 Fig.**

Significant positive correlation between mean CBF and FA values (TFCE corrected p < 0.05) as it is shown in Figure 1 shown for women (in red) and men (in blue) separately. A post-hoc analysis of variance of linear model fits showed that the comparison of the two models (model 1 where CBF is modelled by FA values only and model 2 where CBF values are modelled by FA values and gender) did not differ significantly (p = 0.211, F (36, 37) = 1.62).
