# Supplementary material for: Microstructure and Cerebral Blood Flow within White Matter of the Human Brain: A TBSS Analysis
Source: PLoS One. 2016 Mar 4;11(3):e0150657. doi: 10.1371/journal.pone.0150657 (PMC4778945; doi:10.1371/journal.pone.0150657)
Supplement: S14 Fig — This figure shows the SNR for pure GM, pure WM and for the ASL signal that was extracted for the regions of significant positive correlation between CBF and FA values (regions from Fig 1). The tissue classes were corrected for possible confounding effects originated by partial volume effects. (DOCX) [file pone.0150657.s014.docx]

**SNR signal characteristics**

**
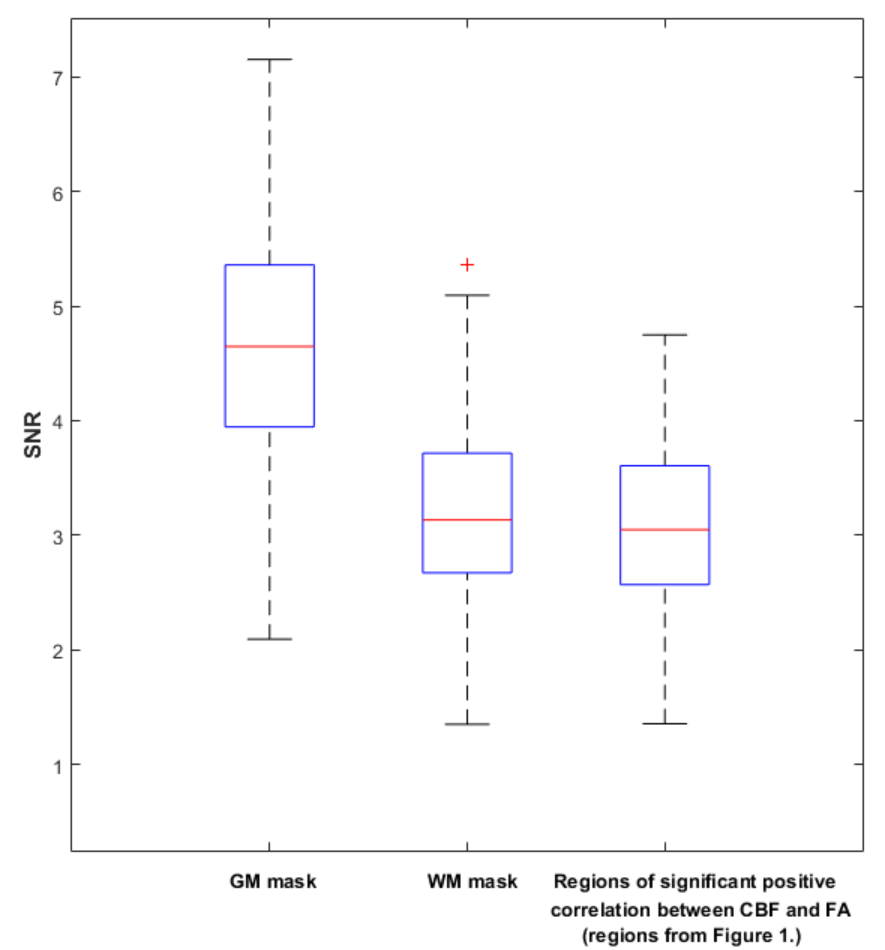
**

**S14 Fig.**

This figure shows the SNR for pure GM, pure WM and for the ASL signal that was extracted for the regions of significant positive correlation between CBF and FA values (regions from Fig 1). The tissue classes were corrected for possible confounding effects originated by partial volume effects.
