# Supplementary material for: Microstructure and Cerebral Blood Flow within White Matter of the Human Brain: A TBSS Analysis
Source: PLoS One. 2016 Mar 4;11(3):e0150657. doi: 10.1371/journal.pone.0150657 (PMC4778945; doi:10.1371/journal.pone.0150657)
Supplement: S15 Fig — This figure shows the difference values from the subtraction between control and label images for pure GM, pure WM and for the ASL signal that was extracted for the regions of significant positive correlation between CBF and FA values (regions from Fig 1). The tissue classes were corrected for possible confounding effects originated by partial volume effects. These difference values are the input parameters for the CBF quantification ΔM in the equation of page 7. (DOCX) [file pone.0150657.s015.docx]

**Control-label difference**


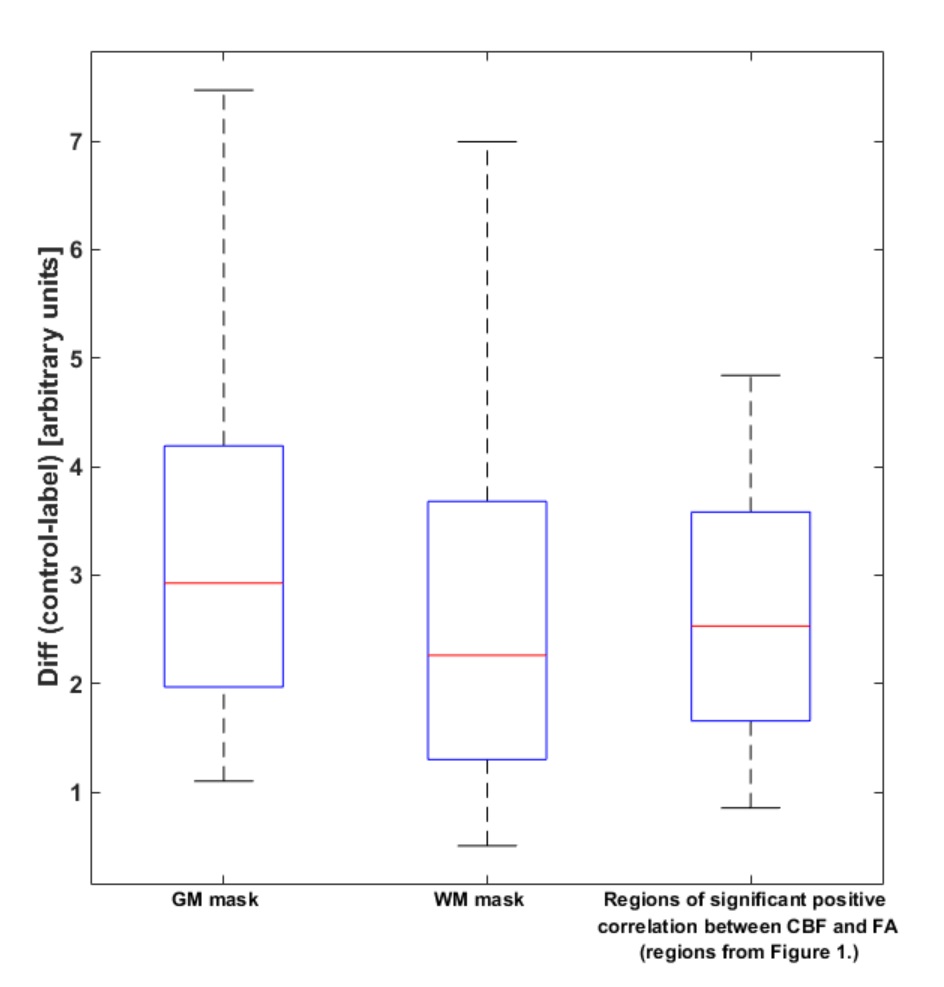


**S15 Fig.**

This figure shows the difference values from the subtraction between control and label images for pure GM, pure WM and for the ASL signal that was extracted for the regions of significant positive correlation between CBF and FA values (regions from Fig 1). The tissue classes were corrected for possible confounding effects originated by partial volume effects. These difference values are the input parameters for the CBF quantification ∆M in the equation of page 7.
