# Supplementary material for: Microstructure and Cerebral Blood Flow within White Matter of the Human Brain: A TBSS Analysis
Source: PLoS One. 2016 Mar 4;11(3):e0150657. doi: 10.1371/journal.pone.0150657 (PMC4778945; doi:10.1371/journal.pone.0150657)
Supplement: S16 Fig — This figure shows the CBF values for the additional measure on five subjects with three different values of post-label delay time (1100 ms, 1525 ms and 1900 ms). The CBF values in deep WM shows week dependency on PLD demonstrating that the extracted CBF values with short PLD are slightly underestimated as compared to CBF values with higher PLD, however. Testing the linear model that CBF estimation depends on PLD yields the slope not to be different from zero [t(38) = 1.155; p = 0.188] (in WM–PVE corrected) and [t(38) = 1.259; p = 0.174] in corpus callosum. (DOCX) [file pone.0150657.s016.docx]

**CBF values for 3 different postlabel delay values**

**
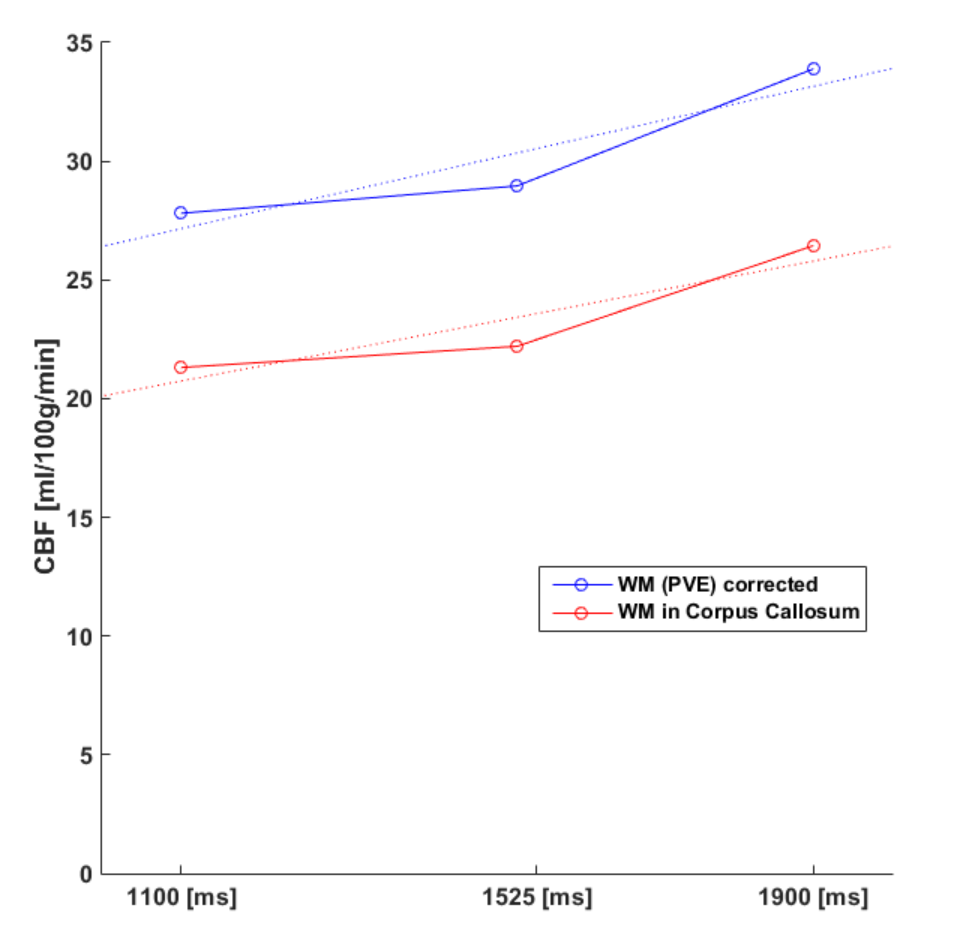
**

**S16 Fig.**

This figure shows the CBF values for the additional measure on five subjects with three different values of post-label delay time (1100 ms, 1525 ms and 1900 ms). The CBF values in deep WM shows week dependency on PLD demonstrating that the extracted CBF values with short PLD are slightly underestimated as compared to CBF values with higher PLD, however. Testing the linear model that CBF estimation depends on PLD yields the slope not to be different from zero [t(38)=1.155; p=0.188] (in WM–PVE corrected) and [t(38)=1.259; p=0.174] in corpus callosum.
