# Supplementary material for: Microstructure and Cerebral Blood Flow within White Matter of the Human Brain: A TBSS Analysis
Source: PLoS One. 2016 Mar 4;11(3):e0150657. doi: 10.1371/journal.pone.0150657 (PMC4778945; doi:10.1371/journal.pone.0150657)
Supplement: S17 Fig — This figure shows the SNR for the additional measure on five subjects with three different values of post-label delay time (1100 ms, 1525 ms and 1900 ms). The SNR values of WM (PVE) shows no dependency on PLD. Testing the linear model that SNR depends on PLD yields the slope not to be different from zero [t(38) = .487; p = 0.627]. (DOCX) [file pone.0150657.s017.docx]

**SNR values for 3 different postlabel delay values**

**
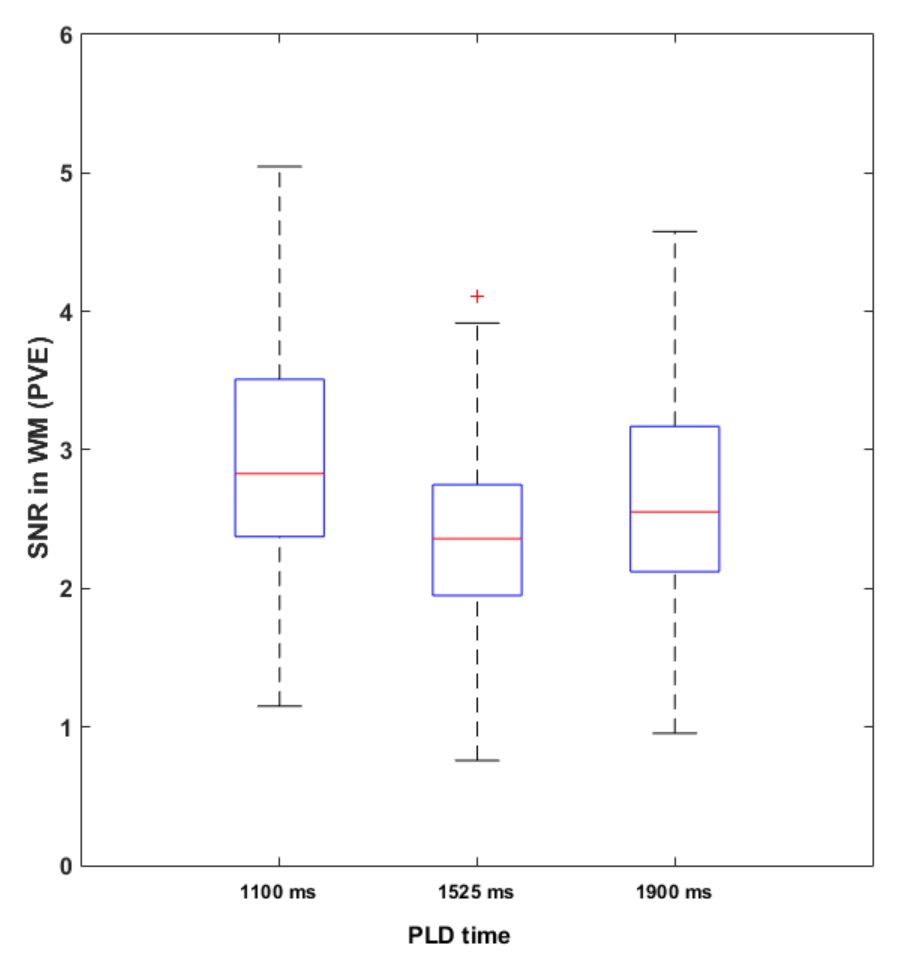
**

**S17 Fig.**

This figure shows the SNR for the additional measure on five subjects with three different values of post-label delay time (1100 ms, 1525 ms and 1900 ms). The SNR values of WM (PVE) shows no dependency on PLD. Testing the linear model that SNR depends on PLD yields the slope not to be different from zero [t(38)=.487; p=0.627].
