# Supplementary material for: Microstructure and Cerebral Blood Flow within White Matter of the Human Brain: A TBSS Analysis
Source: PLoS One. 2016 Mar 4;11(3):e0150657. doi: 10.1371/journal.pone.0150657 (PMC4778945; doi:10.1371/journal.pone.0150657)
Supplement: S1 Table — Relationship between FA and CBF across fiber tracts for each subject. Pearson’s R with p-value is reported. A positive correlation is observed in all subjects—in 21 from 39 subjects the positive correlation is significant. The WM regions are based on the John Hopkins University (JHU)-ICBM-DTI-81 WM labels atlas (48 labels) and the JHU-WM tractography atlasv (20 tracts) in MNI space [58, 59]. Since the ASL image did not cover the lower parts of the brain, the values which are displayed comprise the following 51 WM regions: the genu of corpus callosum, the body of corpus callosum, the splenium of corpus callosum, the bilateral anterior limb of internal capsule, the bilateral posterior limb of internal capsule, the bilateral retrolenticular part of internal capsule, the bilateral anterior corona radiata, the bilateral superior corona radiata, the bilateral posterior corona radiata, the bilateral posterior thalamic radiation, the bilateral sagittal stratum, the bilateral external capsule, the bilateral cingulum (cingulate gyrus), the bilateral fornix (cres) / Stria terminalis, the bilateral superior longitudinal fasciculus, the bilateral superior fronto-occipital fasciculus, the bilateral tapetum, the bilateral anterior thalamic radiation, the bilateral corticospinal tract, the bilateral cingulum (cingulate gyrus), the bilateral cingulum (hippocampus) the forceps major and minor, the bilateral inferior fronto-occipital fasciculus, the bilateral inferior longitudinal fasciculus, the bilateral superior longitudinal fasciculus, the bilateral uncinate fasciculus, the bilateral superior longitudinal fasciculus (temporal part). (DOCX) [file pone.0150657.s018.docx]

**Relationship of CBF and FA across white matter regions for all subjects**

| Subject | dRdPearson’s r | p-value |
| --- | --- | --- |
| S1 * | 0.323 | 0.017 |
| S2 | 0.238 | 0.083 |
| S3 * | 0.382 | 0.005 |
| S4 | 0.258 | 0.062 |
| S5 * | 0.407 | 0.003 |
| S6 | 0.208 | 0.135 |
| S7 * | 0.33 | 0.015 |
| S8 | 0.195 | 0.162 |
| S9 * | 0.294 | 0.031 |
| S10 * | 0.341 | 0.013 |
| S11 | 0.17 | 0.22 |
| S12 * | 0.319 | 0.019 |
| S13 | 0.058 | 0.679 |
| S14 | 0.215 | 0.118 |
| S15 * | 0.37 | 0.006 |
| S16 | 0.109 | 0.433 |
| S17 | 0.146 | 0.293 |
| S18 * | 0.268 | 0.05 |
| S19 * | 0.278 | 0.042 |
| S20 * | 0.376 | 0.005 |
| S21 * | 0.326 | 0.017 |
| S22 * | 0.274 | 0.047 |
| S23 | 0.125 | 0.366 |
| S24 * | 0.318 | 0.019 |
| S25 * | 0.325 | 0.016 |
| S26 * | 0.299 | 0.03 |
| S27 | 0.18 | 0.193 |
| S28 | 0.092 | 0.506 |
| S29 * | 0.549 | <0.001 |
| S30 * | 0.297 | 0.029 |
| S31 * | 0.029 | 0.048 |
| S32 | 0.23 | 0.835 |
| S33 | 0.177 | 0.098 |
| S34 | 0.067 | 0.201 |
| S35 | 0.29 | 0.631 |
| S36 * | 0.387 | 0.035 |
| S37 * | 0.057 | 0.004 |
| S38 | 0.323 | 0.68 |
| S39 | 0.198 | 0.152 |

**S1 Table.**

Relationship between FA and CBF across fiber tracts for each subject. Pearson’s R with p-value is reported. A positive correlation is observed in all subjects - in 21 from 39 subjects the positive correlation is significant. The WM regions are based on the John Hopkins University (JHU)-ICBM-DTI-81 WM labels atlas (48 labels) and the JHU-WM tractography atlasv (20 tracts) in MNI space ([Mazziotta et al., 2001](#_ENREF_2); [Mori et al., 2005](#_ENREF_3)). Since the ASL image did not cover the lower parts of the brain, the values which are displayed comprise the following 51 WM regions: the genu of corpus callosum, the body of corpus callosum, the splenium of corpus callosum, the bilateral anterior limb of internal capsule, the bilateral posterior limb of internal capsule, the bilateral retrolenticular part of internal capsule, the bilateral anterior corona radiata, the bilateral superior corona radiata, the bilateral posterior corona radiata, the bilateral posterior thalamic radiation, the bilateral sagittal stratum, the bilateral external capsule, the bilateral cingulum (cingulate gyrus), the bilateral fornix (cres) / Stria terminalis, the bilateral superior longitudinal fasciculus, the bilateral superior fronto-occipital fasciculus, the bilateral tapetum, the bilateral anterior thalamic radiation, the bilateral corticospinal tract, the bilateral cingulum (cingulate gyrus), the bilateral cingulum (hippocampus) the forceps major and minor, the bilateral inferior fronto-occipital fasciculus, the bilateral inferior longitudinal fasciculus, the bilateral superior longitudinal fasciculus, the bilateral uncinate fasciculus, the bilateral superior longitudinal fasciculus (temporal part).
